# Time Dependent MHD Nano-Second Grade Fluid Flow Induced by Permeable Vertical Sheet with Mixed Convection and Thermal Radiation

**DOI:** 10.1371/journal.pone.0124929

**Published:** 2015-05-11

**Authors:** Muhammad Ramzan, Muhammad Bilal

**Affiliations:** 1 Department of Mathematics, College of Science, Majmaah University, Al-Zulfi, Saudi Arabia; 2 Department of Mathematics, Faculty of Computing, Mohammad Ali Jinnah University, Islamabad Campus, Islamabad, Pakistan; China University of Mining and Technology, CHINA

## Abstract

The aim of present paper is to study the series solution of time dependent MHD second grade incompressible nanofluid towards a stretching sheet. The effects of mixed convection and thermal radiation are also taken into account. Because of nanofluid model, effects Brownian motion and thermophoresis are encountered. The resulting nonlinear momentum, heat and concentration equations are simplified using appropriate transformations. Series solutions have been obtained for velocity, temperature and nanoparticle fraction profiles using Homotopy Analysis Method (HAM). Convergence of the acquired solution is discussed critically. Behavior of velocity, temperature and concentration profiles on the prominent parameters is depicted and argued graphically. It is observed that temperature and concentration profiles show similar behavior for thermophoresis parameter *Νt* but opposite tendency is noted in case of Brownian motion parameter *Νb*. It is further analyzed that suction parameter *S* and Hartman number *Μ* depict decreasing behavior on velocity profile.

## Introduction

Many engineering and industrial applications involve a working fluid that may be active or inactive in its own capacity. The role of this fluid is to transfer energy/heat from one location to other. For a long period, the performance of adequate heat transfer has been a major problem. The introduction of nanofluid as a working fluid has opened the gates of new era in the area of heat transfer. With thermal conductivity more than base fluid and a size of 1–100 nm, nanoparticles are utilized to attain the maximum enhancement in the thermal characteristics under minimum concentrations. The pioneering work of Choi [1] with the declaration that thermal conductivity of base fluid will be doubled by adding the nanoparticles into the base fluid revolutionized the related engineering applications in a variety of directions. These include coolants of nuclear reactors, cancer therapy, safer surgeries and in safety problems related to nuclear reactors. In designing the waste heat removal equipment, nanoparticles play an important role [2]. With both liquid and magnetic properties, magneto nanofluid with its varied biomedical applications like sterilized devices, wound treatment, gastric medications, asthma treatment and elimination of tumors has a vital role in daily life. Some recent studies on nanofluids and magneto nanofluids may be found in the references [3–12] and many therein.

Comprehensive knowledge of non-Newtonian fluids’ flow characteristics is the need of the day because of their vital role in growing industrial and engineering applications. These may include shampoos, soaps, apple sauce, polymeric liquids, tomato paste, ketchup, paints, blood at low shear rate etc. The Navier-Stokes equations are not sufficient to explore the true behavior of such materials. Different types of non-Newtonian fluid models are developed in the past to describe the actual behavior of these liquids. The fluid model which is used in the present investigation is a subclass of differential type non-Newtonian fluids and known as second grade fluid. This fluid model is capable to explore the shear thinning and shear thickening effects. Fetecau et al. [13] studied the unsteady flow of second grade fluid induced due to the time-dependent motion of wall. They provided the exact solutions of this flow analysis by employing Fourier sine transform. Helical flows of second grade fluid between two coaxial cylinders are investigated by Jamil et al. [14]. Here the flow generation is due to inner cylinder motion. Hayat et al. [15] reported two dimensional boundary layer flow of second grade fluid with convective boundary condition via homotopy analysis method. Turkyilmazoglu [16] discussed the dual and triple solutions of MHD second grade non-Newtonian fluid in the presence of slip condition. Heat transfer analysis in viscoelastic non-Newtonian fluid flow is discussed by Ashorynejad et al. [17]. Heat source effect in second grade fluid in the presence of power law heat flux condition is explored by Hayat et al. [18]. Hayat et. al [19] discussed the stratifications and mixed convection radiative flow of Jeffrey fluid over a stretching sheet. But very less approaches have been reported in the presence of nanofluids.

To bridge this gap, we have studied the thermal radiation effects in MHD unsteady flow of second grade nanofluid in the presence of mixed convection. The flow is induced due to the vertical stretching sheet. We developed series solutions of velocity, temperature and nanoparticle concentration via homotopy analysis method (HAM) [20–26]. Graphs are plotted to examine the effects of various physical parameters on the dimensionless temperature and nanoparticle concentration fields. Values of skin-friction coefficient, local Nusselt and Sherwood numbers are computed and discussed. From the literature survey, it is revealed that no such analysis is reported yet.

## Mathematical formulation

**Fig 1:**
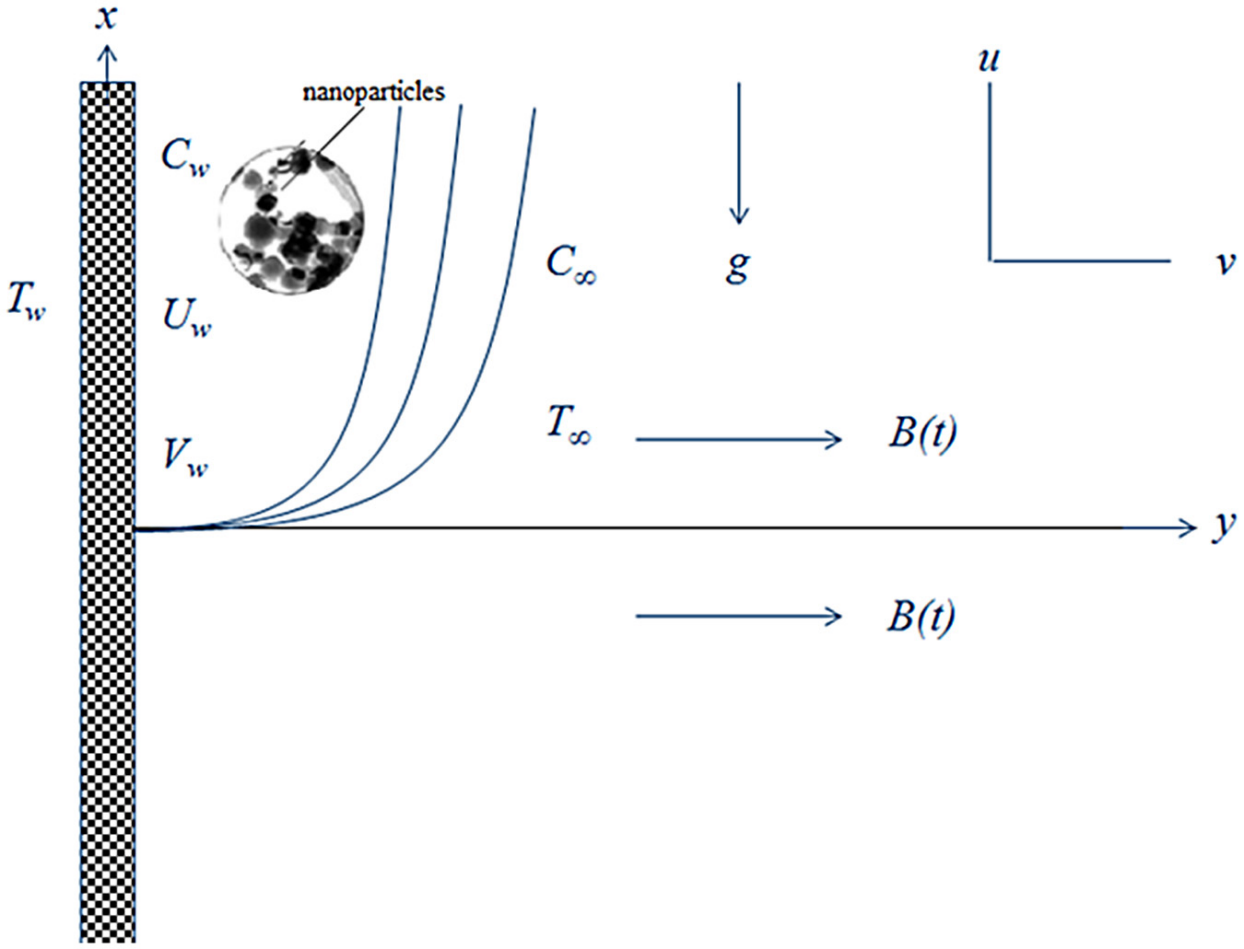
Geometry of the problem.

We consider the magnetohydrodynamics (MHD) and time dependent flow of an incompressible second grade nanofluid over a porous stretching surface. The electrically conducting fluid under the influence of a unsteady magnetic field *B*(*t*) which is applied in a direction normal to the stretching surface. Under the assumption of a small magnetic Reynolds number, the induced magnetic field is negligible. Moreover, heat transfer process is also taken into account. The geometrical configuration of the present flow is shown in Fig 1.

The governing boundary layer equations using above mentioned suppositions and Boussineq’s approximation can be written as:

$$\begin{array}{c} \frac{\partial u}{\partial x}+\frac{\partial v}{\partial y}=0, \end{array}$$ (1)

$$\begin{array}{ccc} \frac{\partial u}{\partial t}+u\frac{\partial u}{\partial x}+v\frac{\partial u}{\partial y} & = & \nu \frac{\partial^{2}u}{\partial y^{2}}+\frac{\alpha_{1}}{\rho }\;(\frac{\partial^{3}u}{\partial t\partial y^{2}}+u\frac{\partial^{3}u}{\partial x\partial y^{2}}+\frac{\partial u}{\partial x}\frac{\partial^{2}u}{\partial y^{2}}+\frac{\partial u}{\partial y}\frac{\partial^{2}v}{\partial y^{2}}+v\frac{\partial^{3}u}{\partial y^{3}})+\\ & & g\beta_{T}\left(T-T_{\infty }\right)+g\beta_{C}\left(C-C_{\infty }\right)-\frac{\sigma B^{2}\;\left(t\right)u}{\rho }, \end{array}$$ (2)

$$\begin{array}{ccc} \rho C_{P}\;(\frac{\partial T}{\partial t}+u\frac{\partial T}{\partial x}+v\frac{\partial T}{\partial y}) & = & \alpha_{1}\;(\frac{\partial^{2}u}{\partial y\partial t}\frac{\partial u}{\partial y}+u\frac{\partial^{2}u}{\partial x\partial y}\frac{\partial u}{\partial y}+v\frac{\partial^{2}u}{\partial y^{2}}\frac{\partial u}{\partial y})+k\frac{\partial^{2}T}{\partial y^{2}}+\\ & & \mu \;{\left(\frac{\partial u}{\partial y}\right)}^{2}-\frac{\partial q_{r}}{\partial y}+\tau \;(D_{B}\frac{\partial C}{\partial y}\frac{\partial T}{\partial y}+\frac{D_{T}}{T_{\infty }}\;{\left(\frac{\partial T}{\partial y}\right)}^{2}), \end{array}$$ (3)

$$\begin{array}{c} \frac{\partial C}{\partial t}+u\frac{\partial C}{\partial x}+v\frac{\partial C}{\partial y}=D_{B}\frac{\partial^{2}C}{\partial y^{2}}+\frac{D_{T}}{T_{\infty }}\frac{\partial^{2}T}{\partial y^{2}}. \end{array}$$ (4)

By using Rosseland approximatoin for radiation we have

$$\begin{array}{c} q_{r}=-\frac{4\sigma^{*}}{3k^{*}}\frac{\partial T^{4}}{\partial y}, \end{array}$$ (5)

in which *q*_*r*_ the radiative heat flux in the *y*-direction, *g* the gravitational acceleration, *T* the fluid temperature, σ* the Stefan-Boltzmann constant, *ν* the kinematic viscosity, *σ* the electrical conductivity, *ρ* the fluid density, *β*_*C*_ and *β*_*T*_ are the concentration and thermal expansion coefficients respectively, *D*_*B*_ and *D*_*T*_ are the Brownian diffusion coefficient and thermophoretic diffusion coefficient, respectively, k* is the mean absorption coefficient, *α*_1_ the second grade parameter and *C*_*p*_ the specific heat and. Since the fluid abide by the second law of thermodynamics and the assumption that the specific Helmholtz free energy is least when the fluid is at a constant temperature then we have *μ* ≥ 0, *α*_1_ ≥ 0, *α*_1_ + *α*_2_ = 0.

Expanding *T* ^4^ in Taylor series about *T*_∞_ and neglecting higher terms, we found

$$\begin{array}{c} T^{4}≊4T_{\infty }^{3}T-3T_{\infty }^{4} \end{array}$$ (6)

By making use of Eqs (5) and (6), Eq (3) has the following form

$$\begin{array}{ccc} \rho C_{P}\;(\frac{\partial T}{\partial t}+u\frac{\partial T}{\partial x}+v\frac{\partial T}{\partial y}) & = & \alpha_{1}\;(\frac{\partial^{2}u}{\partial y\partial t}\frac{\partial u}{\partial y}+u\frac{\partial^{2}u}{\partial x\partial y}\frac{\partial u}{\partial y}+v\frac{\partial^{2}u}{\partial y^{2}}\frac{\partial u}{\partial y})+\\ & & \mu \;{\left(\frac{\partial u}{\partial y}\right)}^{2}-\frac{\partial }{\partial y}\;(\frac{16\sigma^{*}T_{\infty }^{3}}{3k^{*}}+k)\;\frac{\partial T}{\partial y}+\\ & & \tau \;(D_{B}\frac{\partial C}{\partial y}\frac{\partial T}{\partial y}+\frac{D_{T}}{T_{\infty }}\;{\left(\frac{\partial T}{\partial y}\right)}^{2}). \end{array}$$ (7)

The imposed boundary conditions are given below

$$\begin{array}{c} u=U_{w},\;\;v=V_{w},\;\;T=T_{w},\;C=C_{w}\;\;at\;\;y=0, \end{array}$$ (8)

$$\begin{array}{c} u\to 0,\;\;\;T\to T_{\infty },\;C\;\to C_{\infty }\;\;\;\;as\;y\to \infty , \end{array}$$ (9)

where

$$\begin{array}{c} V_{w}=-\frac{v_{0}}{{\left(1-ct\right)}^{1/2}}, \end{array}$$ (10)

is the mass transfer at surface with *V*_*w*_ < 0 for suction and *V*_*w*_ > 0 for injection. Moreover, the surface temperature *T*_*w*_(*x*, *t*), stretching velocity *U*_*w*_(*x*, *t*) and the value of nanoparticle volume fraction *C*_*w*_(*x*, *t*) are given by:

$$\begin{array}{ccc} U_{w}\;\left(x,t\right) & = & \frac{ax}{1-ct},\;\;T_{w}\;\left(x,t\right)=T_{\infty }+T_{0}\frac{ax}{2\nu \;{\left(1-ct\right)}^{2}},\\ C_{w}\;\left(x,t\right) & = & C_{\infty }+C_{0}\frac{ax}{2\nu \;{\left(1-ct\right)}^{2}}, \end{array}$$ (11)

with *a* and *c* are the constants with *a* ≥ 0 and *c* > 0 (with *ct* < 1), and time^−1^ is the dimension for both *a* and *c*. We select unsteady magnetic field of the form *B*(*t*) = *B*_0_(1−*ct*)^−1/2^

Similarity transformation for the present case is given below

$$\begin{array}{ccc} \eta & = & \sqrt{\frac{U_{w}}{\nu x}}y,\;\;\;\;\;\;\;\psi =\sqrt{\nu xU_{w}}f\;(\eta ),\;\\ \;\theta \;(\eta ) & = & \frac{T-T_{\infty }}{T_{w}-T_{\infty }},\;\phi \;(\eta )=\frac{C-C_{\infty }}{C_{w}-C_{\infty }}, \end{array}$$ (12)

and the velocity components

$$\begin{array}{c} u=\frac{\partial \psi }{\partial y},\;\;\;v=-\frac{\partial \psi }{\partial x}, \end{array}$$ (13)

identically satisfies Eq (1) with stream function *ψ* while Eqs (2)–(4) and (7)–(9) are converted into the following form

$$\begin{array}{ccc} & & f^{\prime \prime \prime }-f^{\prime 2}+ff^{\prime \prime }-A\;(f^{\prime }+\frac{\eta }{2}f^{\prime \prime })-M^{2}f^{\prime }+\lambda \;(\theta +N\phi )+\\ & & +\alpha \;(2f^{\prime }f^{\prime \prime \prime }-f^{\prime \prime 2}-ff^{\prime \prime \prime \prime }+A\;(2f^{\prime \prime \prime }+\frac{\eta }{2}f^{\prime \prime \prime \prime }))=0, \end{array}$$ (14)

$$\begin{array}{ccc} & & (1+\frac{4}{3}R_{d})\;\theta^{\prime \prime }+\mathrm{Pr}(f\theta^{\prime }-f^{\prime }\theta )+\mathrm{Pr}Ec\;{\left(f^{\prime \prime }\right)}^{2}-\mathrm{Pr}\frac{A}{2}\;(\eta \theta^{\prime }+4\theta )+\\ & & \mathrm{Pr}Ec\alpha \;(\frac{A}{2}\;(3\;{\left(f^{\prime \prime }\right)}^{2}+\eta f^{\prime \prime }f^{\prime \prime \prime })+f^{\prime }f^{\prime \prime 2}-ff^{\prime \prime }f^{\prime \prime \prime })+\mathrm{Pr}\;(N_{b}\theta^{\prime }\phi^{\prime }+N_{t}\theta^{\prime 2})=0, \end{array}$$ (15)

$$\begin{array}{c} \phi^{\prime \prime }+(\frac{N_{t}}{N_{b}})\;\theta^{\prime \prime }+\mathrm{Pr}Le\;(f\phi^{\prime }-f^{\prime }\phi )-\mathrm{Pr}LeA\;(2\phi +\frac{\eta }{2}\phi^{\prime })=0, \end{array}$$ (16)

$$\begin{array}{ccc} f\;(0) & = & S,\;\;f^{\prime }\left(0\right)=1,\;\;\theta \;(0)=1,\;\;\phi \;\left(0\right)=1,\\ f^{\prime }\left(\infty \right) & \to & 0,\;f^{{}^{\prime }\prime }\;\left(\infty \right)\to 0,\;\;\;\theta \;(\infty )\to 0,\;\phi \;\left(\infty \right)\to 0. \end{array}$$ (17)

Here *A* = *a*/*c* is the unsteadiness parameter, *α* = *bα*_1_/*μ* (1−*ct*), (with *ct* < 1) is the second grade dimensionless parameter, *G*_*r*_*x*__ = *gβ* (*T*_*w*_−*T*_∞_)*x* ^3^/*ν* ^2^ is the Grashof number, $\lambda =Gr_{x}/{\text{Re}}_{x}^{2}$ is mixed convection parameter, Re_*x*_ = *u*_*w*_ *x*/*ν* is the local Reynold number

$$\begin{array}{c} \lambda =\frac{G_{r_{x}}}{{Re}_{x}^{2}}\;(=\frac{g\beta \;(T_{w}-T_{\infty })\;x^{3}/\nu^{2}}{u_{w}^{2}x^{2}/\nu^{{}_{2}}}),\;\;\; \end{array}$$ (18)

$\mathrm{Pr}=\frac{\mu c_{p}}{k}$ is the Prandtl number, $R_{D}=\frac{4\sigma^{*}T_{\infty }^{3}}{3k^{*}k}$ is the radiation parameter, $Ec=\frac{u_{w}^{2}}{c_{p}\left(T_{w}-T_{\infty }\right)}$ is the Eckert number, $N_{b}=\frac{\tau D_{B}}{\nu }(C_{w}-C_{\infty })$ is the Brownian motion parameter, $Nt=\frac{D_{T}}{T_{\infty }}\frac{\tau }{\nu }\;(T_{w}-T_{\infty })$ is the thermophoresis, $Le=\frac{\alpha }{D_{B}}$ is the Lewis number.

The Skin friction coefficient, local Nusselt and local Sherwood numbers are given by the expressions

$$\begin{array}{c} C_{f}=\frac{\tau_{w}}{\rho u_{w}^{2}},\;\;\;\;\;\;Nu_{x}=\frac{xq_{w}}{k\;(T_{w}-T_{\infty })},\;\;\;\;Sh=\;\frac{x\;j_{w}}{D_{B}\;\left(C_{w}-C_{\infty }\right)}\text{,}\; \end{array}$$ (19)

where the skin friction *τ*_*w*_ and wall heat flux *q*_*w*_ and the concentration flux *j*_*w*_ are defined as

$$\begin{array}{ccc} \tau_{w} & = & {\left(\mu \frac{\partial u}{\partial y}+\alpha_{1}\;(\frac{\partial^{2}u}{\partial y\partial t}+2\frac{\partial u}{\partial x}\frac{\partial u}{\partial y}+u\frac{\partial^{2}u}{\partial x\partial y}+v\frac{\partial^{2}u}{\partial y^{2}})\right)}_{y=0},\\ \;\;\;\;\;q_{w} & = & -k\;{\left(\frac{\partial T}{\partial r}\right)}_{y=0},\;\;j_{w}=-D_{B}\;{\left(\frac{\partial C}{\partial r}\right)}_{r=0}. \end{array}$$ (20)

Dimensionless forms of skin friction coefficient, local Nusselt and local Sherwood numbers are

$$\begin{array}{ccc} C_{f}{Re}_{x}^{1/2} & = & {\left(f^{\prime \prime }(\eta )+\alpha \;(\begin{array}{c} 3f^{\prime }(\eta )f^{\prime \prime }(\eta )-f(\eta )f^{\prime \prime \prime }(\eta )+\\ \frac{A}{2}\;(3f^{\prime \prime }(\eta )+\eta f^{\prime \prime \prime }(\eta )) \end{array})\right)}_{\eta =0},\\ N_{u_{x}}{Re}_{x}^{-1/2} & = & -\theta^{\prime }(0),\;\;\;\;\;\;\;\;\;\;\;\;\;\;\;\;\;Sh{Re}_{x}^{-1/2}=-\phi^{\prime }(0), \end{array}$$ (21)

where Re_*z*_ = *w*_*e*_ *z*/*ν* is the Reynolds number.

## Homotopic solutions

The initial guesses and the auxiliary linear operators are essential for the homotopic solutions. The initial guesses and the auxiliary linear operators for the present flow problems are

$$\begin{array}{c} f_{0}\;(\eta )=S+(1-\exp(-\eta )),\;\;\;\;\theta_{0}\;(\eta )=\exp(-\eta ),\;\;\;\;\phi_{0}\;(\eta )=\exp(-\eta ). \end{array}$$ (22)

$$\begin{array}{c} {𝓛}_{f}\;(\eta )=\frac{d^{3}f}{d\eta^{3}}-\frac{df}{d\eta },\;{𝓛}_{\theta }(\eta )=\frac{d^{2}\theta }{d\eta^{2}}-\theta ,\;{𝓛}_{\phi }(\eta )=\frac{d^{2}\phi }{d\eta^{2}}-\phi . \end{array}$$ (23)

The auxiliary linear operators have the following properties

$$\begin{array}{c} {𝓛}_{f}[C_{1}+C_{2}\exp\left(\eta \right)+C_{3}\exp\left(-\eta \right)]=0, \end{array}$$ (24)

$$\begin{array}{c} {𝓛}_{\theta }[C_{4}\exp\left(\eta \right)+C_{5}\exp\left(-\eta \right)]=0, \end{array}$$ (25)

$$\begin{array}{c} {𝓛}_{\phi }[C_{6}\exp\left(\eta \right)+C_{7}\exp\left(-\eta \right)]=0, \end{array}$$ (26)

where *C*_*i*_ (*i* = 1−7) are the arbitrary constants. The zeroth and *m*th order deformation problems are stated below.

### Zeroth-order problem

The problems at zeroth order deformation can be expressed as

$$\begin{array}{c} (1-p){𝓛}_{f}[\hat{f}(\eta ;p)-f_{0}\;(\eta )]=p\hbar_{f}{𝓝}_{f}[\hat{f}\;(\eta ;p),\hat{\theta }\;(\eta ;p),\hat{\phi }\;(\eta ;p)], \end{array}$$ (27)

$$\begin{array}{c} (1-p){𝓛}_{\theta }[\hat{\theta }\;(\eta ;p)-\theta_{0}\;(\eta )]=p\hbar_{\theta }{𝓝}_{\theta }[\hat{\theta }\;(\eta ;p),\hat{f}\;(\eta ;p),\hat{\phi }(\eta ;p)], \end{array}$$ (28)

$$\begin{array}{c} (1-p){𝓛}_{\phi }[\hat{\phi }\;(\eta ;p)-\phi_{0}\;(\eta )]=p\hbar_{\phi }{𝓝}_{\phi }[\hat{\phi }\;(\eta ;p),\hat{f}\;(\eta ;p),\hat{\theta }(\eta ;p)], \end{array}$$ (29)

$$\begin{array}{c} {\hat{f}(\eta ;p)|}_{\eta =0}=S,\;\;\;\;{\frac{\partial \hat{f}(\eta ;p)}{\partial \eta }|}_{\eta =0}=1,\;\;\;\;{\frac{\partial \hat{f}(\eta ;p)}{\partial \eta }|}_{\eta =\infty }=1, \end{array}$$ (30)

$$\begin{array}{c} {\hat{\theta }\;(\eta ;p)|}_{\eta =0}=1,\;\;\;\;\;\;{\hat{\theta }\;(\eta ;p)|}_{\eta =\infty }=0, \end{array}$$ (31)

$$\begin{array}{c} {\hat{\phi }\;(\eta ;p)|}_{\eta =0}=1,\;\;\;\;\;\;{\hat{\phi }\;(\eta ;p)|}_{\eta =\infty }=0, \end{array}$$ (32)

$$\begin{array}{ccc} {𝓝}_{f}\;(\hat{f}\;(\eta ,p),\hat{\theta }\;(\eta ;p),\hat{\phi }\;(\eta ;p)) & = & \frac{\partial^{3}\hat{f}\;(\eta ;p)}{\partial \eta^{3}}+\hat{f}(\eta ;p)\frac{\partial^{2}\hat{f}(\eta ;p)}{\partial \eta^{2}}-{\left(\frac{\partial \hat{f}(\eta ;p)}{\partial \eta }\right)}^{2}\\ & & -M^{2}\frac{\partial \hat{f}(\eta ;p)}{\partial \eta }-A\;(\frac{\eta }{2}\frac{\partial^{2}\hat{f}(\eta ;p)}{\partial \eta^{2}}+\frac{\partial \hat{f}(\eta ;p)}{\partial \eta })+\\ & & \alpha \;(\begin{array}{c} -{\left(\frac{\partial^{2}\hat{f}(\eta ;p)}{\partial \eta^{2}}\right)}^{2}-A(2\frac{\partial^{3}\hat{f}(\eta ;p)}{\partial \eta^{3}}+\frac{\eta }{2}\frac{\partial^{4}\hat{f}(\eta ;p)}{\partial \eta^{4}})\\ +2\frac{\partial \hat{f}(\eta ;p)}{\partial \eta }\frac{\partial^{3}\hat{f}(\eta ;p)}{\partial \eta^{3}}-\frac{\partial \hat{f}(\eta ;p)}{\partial \eta }\frac{\partial^{4}\hat{f}(\eta ;p)}{\partial \eta^{4}} \end{array})+\\ & & \lambda (\hat{\theta }\;(\eta ;p)+N\hat{\phi }\;(\eta ;p))+\lambda \hat{\theta }\;(\eta ;p), \end{array}$$ (33)

$$\begin{array}{ccc} {𝓝}_{\theta }(\hat{\theta }(\eta ;p),\hat{f}(\eta ;p),\hat{\phi }(\eta ;p)) & = & (1+\frac{4}{3}R_{d})\;\frac{\partial^{2}\hat{\theta }\left(\eta ,p\right)}{\partial \eta^{2}}+\\ & & \mathrm{Pr}(\hat{f}(\eta ;p)\;\frac{\partial \hat{\theta }\;(\eta ;p)}{\partial \eta }-\frac{\partial \hat{f}\;(\eta ;p)}{\partial \eta }\hat{\theta }\left(\eta ,p\right))+\\ & & \mathrm{Pr}Ec\;{\left(\frac{\partial^{2}\hat{f}\;(\eta ;p)}{\partial \eta^{2}}\right)}^{2}-\mathrm{Pr}\frac{A}{2}\;(\eta \frac{\partial \hat{\theta }\;(\eta ;p)}{\partial \eta }+4\hat{\theta }\;(\eta ;p))+\\ & & \mathrm{Pr}Ec\alpha \;(\begin{array}{c} \frac{A}{2}\;(3\;{\left(\frac{\partial^{2}\hat{f}\;(\eta ;p)}{\partial \eta^{2}}\right)}^{2}+\eta \frac{\partial^{2}\hat{f}\;(\eta ;p)}{\partial \eta^{2}}\frac{\partial^{3}\hat{f}(\eta ;p)}{\partial \eta^{3}})\\ +\frac{\partial \hat{f}(\eta ;p)}{\partial \eta }{\left(\frac{\partial^{2}\hat{f}(\eta ;p)}{\partial \eta^{2}}\right)}^{2}\\ -\hat{f}(\eta ;p)\frac{\partial^{2}\hat{f}(\eta ;p)}{\partial \eta^{2}}\frac{\partial^{3}\hat{f}(\eta ;p)}{\partial \eta^{3}} \end{array})\\ & & +\mathrm{Pr}(N_{b}\frac{\partial \hat{\theta }(\eta ;p)}{\partial \eta }\frac{\partial \hat{\phi }\left(\eta ,p\right)}{\partial \eta }+N_{t}{\left(\frac{\partial \hat{\theta }\left(\eta ,p\right)}{\partial \eta }\right)}^{2}), \end{array}$$ (34)

$$\begin{array}{ccc} {𝓝}_{\phi }(\hat{\phi }(\eta ;p),\hat{f}(\eta ;p),\hat{\theta }(\eta ;p)) & = & \frac{\partial^{2}\hat{\phi }\left(\eta ,p\right)}{\partial \eta^{2}}+\mathrm{Pr}Le\;(\hat{f}(\eta ;p)\frac{\partial \hat{\phi }(\eta ;p)}{\partial \eta }-\frac{\partial \hat{f}(\eta ;p)}{\partial \eta }\hat{\phi }\left(\eta ,p\right))\\ & & -\mathrm{Pr}Le\;(A\;(2\hat{\phi }(\eta ;p)+\frac{\eta }{2}\frac{\partial \hat{\phi }\left(\eta ,p\right)}{\partial \eta }))+\frac{N_{t}}{N_{b}}\frac{\partial^{2}\hat{\theta }\left(\eta ,p\right)}{\partial \eta^{2}}. \end{array}$$ (35)

For p = 0 and p = 1, we have

$$\begin{array}{c} \hat{f}\;(\eta ;0)=f_{0}\;(\eta ),\;\;\;\hat{f}\;(\eta ;1)=f\;(\eta ), \end{array}$$ (36)

$$\begin{array}{c} \hat{\theta }\;(\eta ;0)=\theta_{0}\;(\eta ),\;\;\;\hat{\theta }\;(\eta ;1)=\theta \;(\eta ), \end{array}$$ (37)

$$\begin{array}{c} \hat{\phi }\;(\eta ;0)=\phi_{0}\;(\eta ),\;\;\;\hat{\phi }\;(\eta ;1)=\phi \;(\eta ), \end{array}$$ (38)

and when p increases from 0 to 1, then $\hat{f}\;\left(\eta ;p\right),\hat{\theta }\;\left(\eta ;p\right)$ and $\hat{\phi }\;\left(\eta ;p\right)$ changes from *f*_0_(*η*), *θ*_0_(*η*) and *ϕ*_0_(*η*), the initial guess, to *f* (*η*), *θ* (*η*) and *ϕ* (*η*), the final solutions, respectively. Expanding $\hat{f}\left(\eta ;p\right),\hat{\theta }\left(\eta ;p\right)$ and $\hat{\phi }\left(\eta ;p\right)$ we have

$$\begin{array}{c} \hat{f}(\eta ;p)=f_{0}\;(\eta )+\sum_{m=1}^{\infty }f_{m}\;(\eta )p^{m}, \end{array}$$ (39)

$$\begin{array}{c} \hat{\theta }\;(\eta ;p)=\theta_{0}\;(\eta )+\sum_{m=1}^{\infty }\theta_{m}\;(\eta )p^{m}, \end{array}$$ (40)

$$\begin{array}{c} \hat{\phi }\;(\eta ;p)=\phi_{0}\;(\eta )+\sum_{m=1}^{\infty }\phi \;(\eta )p^{m}. \end{array}$$ (41)

### *m*th-order deformation problems

$$\begin{array}{c} {𝓛}_{f}[f_{m}\;(\eta )-\chi_{m}f_{m-1}\;(\eta )]=\hbar_{f}{𝓡}_{m}^{f}\;(\eta ), \end{array}$$ (42)

$$\begin{array}{c} {𝓛}_{\theta }[\theta_{m}\;(\eta )-\chi_{m}\theta_{m-1}\;(\eta )]=\hbar_{\theta }{𝓡}_{m}^{\theta }\;(\eta ), \end{array}$$ (43)

$$\begin{array}{c} {𝓛}_{\phi }[\phi_{m}\;(\eta )-\chi_{m}\phi_{m-1}\;(\eta )]=\hbar_{\phi }{𝓡}_{m}^{\phi }\;(\eta ), \end{array}$$ (44)

$$\begin{array}{c} f_{m}\;(0)=f_{m}^{\prime }\;(0)=f_{m}^{\prime }\;(\infty )=0, \end{array}$$ (45)

$$\begin{array}{c} \theta_{m}\;(0)=\theta_{m}\;(\infty )=0, \end{array}$$ (46)

$$\begin{array}{c} \phi_{m}\;(0)=\phi_{m}\;(\infty )=0, \end{array}$$ (47)

$$\begin{array}{ccc} {𝓡}_{m}^{f}\;(\eta ) & = & f_{m-1}^{\prime \prime \prime }-A\;(f_{m-1}^{\prime }+\frac{1}{2}\eta f_{m-1}^{\prime \prime })+\alpha A\;(2f_{m-1}^{\prime \prime \prime }+\frac{1}{2}\eta f_{m-1}^{\prime \prime \prime \prime })-M^{2}f_{m-1}^{\prime }+\\ & & \sum_{k=0}^{m-1}[f_{m-1-k}f_{k}^{\prime \prime }-f_{m-1-k}^{\prime }f_{k}^{\prime }+\alpha \;(2f_{m-1-k}f_{k}^{\prime \prime \prime }-f_{m-1-k}^{\prime \prime }f_{k}^{\prime \prime }-f_{m-1-k}f_{k}^{\prime \prime \prime \prime })]\\ & & +\lambda \;(\theta_{m-1-k}+N\phi_{m-1-k}), \end{array}$$ (48)

$$\begin{array}{ccc} {𝓡}_{m}^{\theta }\;(\eta ) & = & \left(1+\frac{4}{3}R_{d})\;\theta_{m-1}^{\prime \prime }-\mathrm{Pr}\frac{A}{2}\;(\eta \theta_{m-1}^{\prime }+4\theta_{m-1-k})+\mathrm{Pr}\sum_{k=0}^{m-1}(f_{m-1-k}\theta_{k}^{\prime }-f_{m-1-k}^{\prime }\theta_{k}\right)\\ & & +\mathrm{Pr}Ec\;[\sum_{k=0}^{m-1}\;[f_{m-1-k}^{\prime }f_{k}^{\prime \prime }+\alpha \;(\begin{array}{c} \frac{A}{2}\;(3f_{m-1-k}^{\prime \prime }f_{k}^{\prime \prime }+\eta f_{m-1-k}^{\prime \prime }f_{k}^{\prime \prime \prime })\\ +f_{m-1-k}^{\prime }\sum_{k=0}^{m-1}f_{k-l}^{\prime \prime }f_{l}^{\prime \prime }-f_{m-1-k}\sum_{k=0}^{m-1}f_{k-l}^{\prime \prime }f_{l}^{\prime \prime \prime } \end{array})]]\\ & & \mathrm{Pr}(N_{b}\sum_{k=0}^{m-1}(\theta_{m-1-k}^{\prime }\phi_{k}-\theta_{m-1-k}^{\prime }\theta_{k}^{\prime })+N_{t}\sum_{k=0}^{m-1}(f_{m-1-k}\theta_{k}^{\prime }-f_{m-1-k}^{\prime }\theta_{k})), \end{array}$$ (49)

$$\begin{array}{ccc} {𝓡}_{m}^{\phi }\;(\eta ) & = & \phi_{m-1}^{\prime \prime }+2\gamma \phi_{m-1}^{\prime }+\mathrm{Pr}Le\sum_{k=0}^{m-1}(f_{m-1-k}\phi_{k}^{\prime }-f_{m-1-k}^{\prime }\phi_{k})+\frac{N_{t}}{N_{b}}\theta_{m-1}^{\prime \prime }-\\ & & \mathrm{Pr}Le\;[A\;(2\phi_{m-1-k}+\frac{\eta }{2}\phi_{m-1}^{\prime })], \end{array}$$ (50)

$$\begin{array}{c} \chi_{m}=\{\begin{array}{c} 0,\;\;\;m\leq 1\\ 1,\;\;\;m>1 \end{array}. \end{array}$$ (51)

The general solutions of the Equations are

$$\begin{array}{c} f_{m}\;(\eta )=f_{m}^{*}\;(\eta )+C_{1}+C_{2}\exp\;(\eta )+C_{3}\exp\;(\eta ), \end{array}$$ (52)

$$\begin{array}{c} \theta_{m}\;(\eta )=\theta_{m}^{*}\;(\eta )+C_{4}\exp\;(\eta )+C_{5}\exp\;(\eta ), \end{array}$$ (53)

$$\begin{array}{c} \phi_{m}\;(\eta )=\phi_{m}^{*}\;(\eta )+C_{6}\exp\;(\eta )+C_{7}\exp\;(\eta ). \end{array}$$ (54)

For *p* = 0 and *p* = 1, we have

$$\begin{array}{c} \hat{f}\;(\eta ;0)=f_{0}\;(\eta ),\;\;\hat{f}\;(\eta ;1)=f\;(\eta ), \end{array}$$ (55)

$$\begin{array}{c} \hat{\theta }\;(\eta ;0)=\theta_{0}\;(\eta ),\;\;\;\hat{\theta }\;(\eta ;1)=\theta \;(\eta ), \end{array}$$ (56)

$$\begin{array}{c} \hat{\phi }\;(\eta ;0)=\phi_{0}\;(\eta ),\;\;\;\hat{\phi }\;(\eta ;1)=\phi \;(\eta ), \end{array}$$ (57)

and with the variation of *p* from 0 to 1, $\hat{f}\left(\eta ;p\right),$ $\hat{\theta }\left(\eta ;p\right)\;$ and $\hat{\phi }\left(\eta ;p\right)\;$ vary from the initial solutions *f*_0_(*η*), *θ*_0_(*η*) and *ϕ*_0_(*η*) to the final solutions *f* (*η*), *θ* (*η*) and *ϕ* (*η*) respectively. By Taylor’s series we have

$$\begin{array}{c} \hat{f}\;(\eta ;p)=f_{0}\;(\eta )+\sum_{m=1}^{\infty }f_{m}\;(\eta )p^{m},\;\;\;\;f_{m}\;(\eta )={\frac{1}{m!}\frac{\partial^{m}\hat{f}\;(\eta ;p)}{\partial p^{m}}|}_{p=0}, \end{array}$$ (58)

$$\begin{array}{c} \hat{\theta }\;(\eta ;p)=\theta_{0}\;(\eta )+\sum_{m=1}^{\infty }\theta_{m}\;(\eta )p^{m},\;\;\;\;\;\;\;\theta_{m}\;(\eta )={\frac{1}{m!}\frac{\partial^{m}\hat{\theta }\;(\eta ;p)}{\partial p^{m}}|}_{p=0}, \end{array}$$ (59)

$$\begin{array}{c} \hat{\phi }\;(\eta ;p)=\phi_{0}\;(\eta )+\sum_{m=1}^{\infty }\phi_{m}\;(\eta )p^{m},\;\;\;\;\;\;\;\phi_{m}\;(\eta )={\frac{1}{m!}\frac{\partial^{m}\hat{\phi }\;(\eta ;p)}{\partial p^{m}}|}_{p=0}. \end{array}$$ (60)

The value of auxiliary parameter is chosen in such a way that the series (43)–(45) converge at *p* = 1 i.e.,

$$\begin{array}{c} f\;(\eta )=f_{0}\;(\eta )+\sum_{m=1}^{\infty }f_{m}\;(\eta ), \end{array}$$ (61)

$$\begin{array}{c} \theta \;(\eta )=\theta_{0}\;(\eta )+\sum_{m=1}^{\infty }\theta_{m}\;(\eta ), \end{array}$$ (62)

$$\begin{array}{c} \phi \;(\eta )=\phi_{0}\;(\eta )+\sum_{m=1}^{\infty }\phi_{m}\;(\eta ). \end{array}$$ (63)

The general solutions (*f*_*m*_, *θ*_*m*_, *ϕ*_*m*_) of Eqs (30)–(32) in terms of special solutions $\left(f_{m}^{*},\theta_{m}^{*},\phi_{m}^{*}\right)$ are given by

$$\begin{array}{c} f_{m}\;(\eta )=f_{m}^{⋆}\;(\eta )+A_{1}+A_{2}e^{\eta }+A_{3}e^{-\eta }, \end{array}$$ (64)

$$\begin{array}{c} \theta_{m}\;(\eta )=\theta_{m}^{⋆}\;(\eta )+A_{4}e^{\eta }+A_{5}e^{-\eta }, \end{array}$$ (65)

$$\begin{array}{c} \phi_{m}\;(\eta )=\phi_{m}^{⋆}\;(\eta )+A_{6}e^{\eta }+A_{7}e^{-\eta }, \end{array}$$ (66)

in above expressions, $f_{m}^{*}\left(\eta \right),\theta_{m}^{*}\left(\eta \right)$ and $\phi_{m}^{*}\left(\eta \right)$ denotes the special functions and the constants *A*_*i*_ (*i* = 1−7) through the boundary conditions have the values

$$\begin{array}{ccc} A_{2} & = & A_{4}=A_{6}=0,\;\;\;\;\;\;A_{3}={\frac{\partial f_{m}^{⋆}\;(\eta )}{\partial \eta }|}_{\eta =0}\text{,}\;\;\;\;\;\;A_{1}=-A_{3}-f_{m}^{⋆}(0)\text{,}\;\;\;\;\;\;\;\\ A_{5} & = & -\theta_{m}^{*}(0),\;A_{7}=-\phi_{m}^{*}\;(0). \end{array}$$ (67)

### Convergence of solution

**Fig 2:**
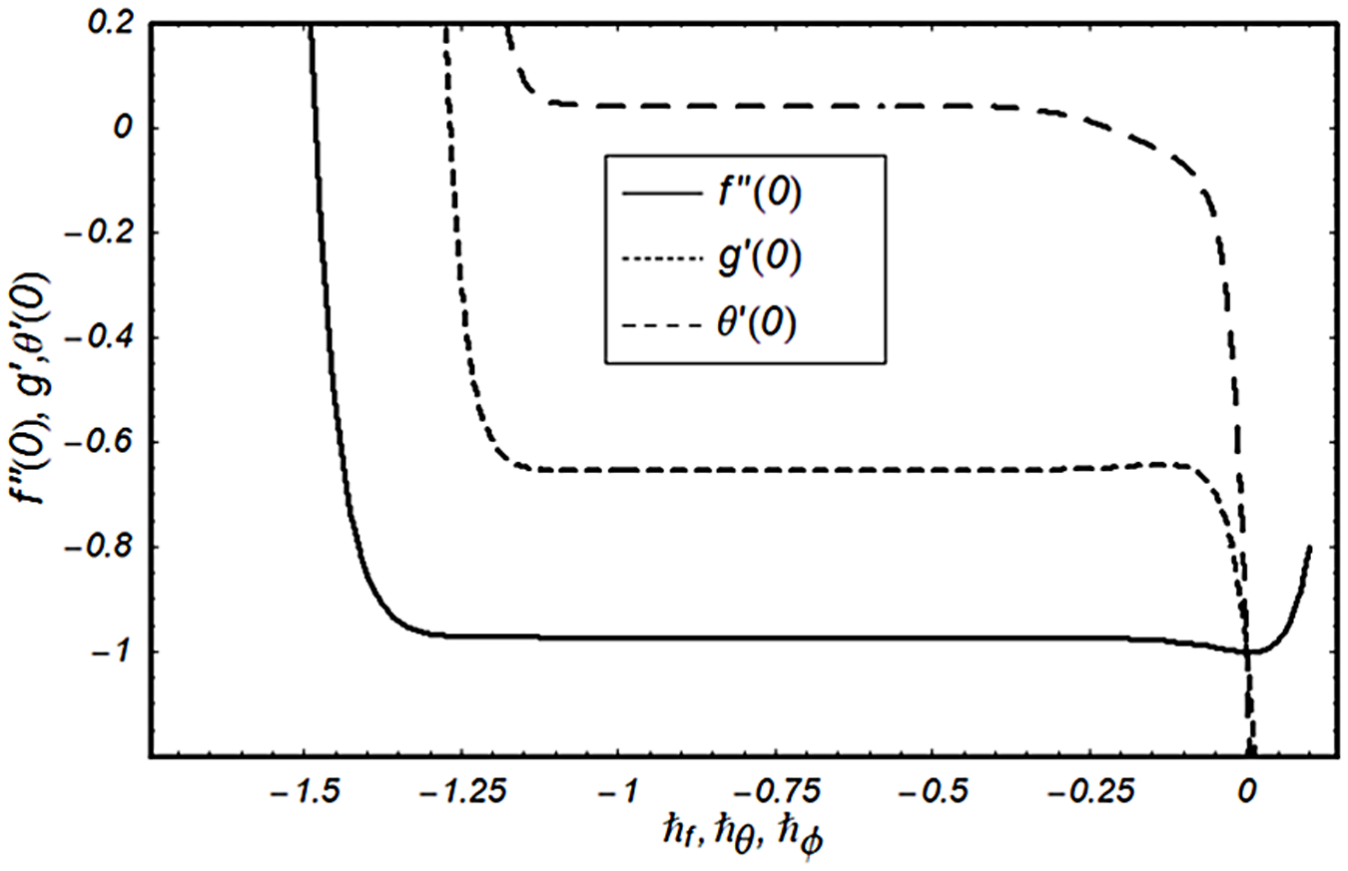
ℏ-curve for function *f*, *θ*, *ϕ*.

To find the meaningful series solutions of momentum, energy and concentration equations, the convergence region is essential to determine. Convergence region of the series solutions depend upon the auxiliary parameter ℏ. Therefore we have plotted the ℏ-curves in the Fig 2. The tolerable range for admissible values of the auxiliary parameters ℏ_*f*_, ℏ_*θ*_ and ℏ_*ϕ*_ are −1.1 ≤ ℏ_*f*_ ≤ −0.5, −1.1 ≤ ℏ_*θ*_ ≤ −0.5 and −1.1 ≤ ℏ_*ϕ*_ ≤ −0.45.

**Fig 3:**
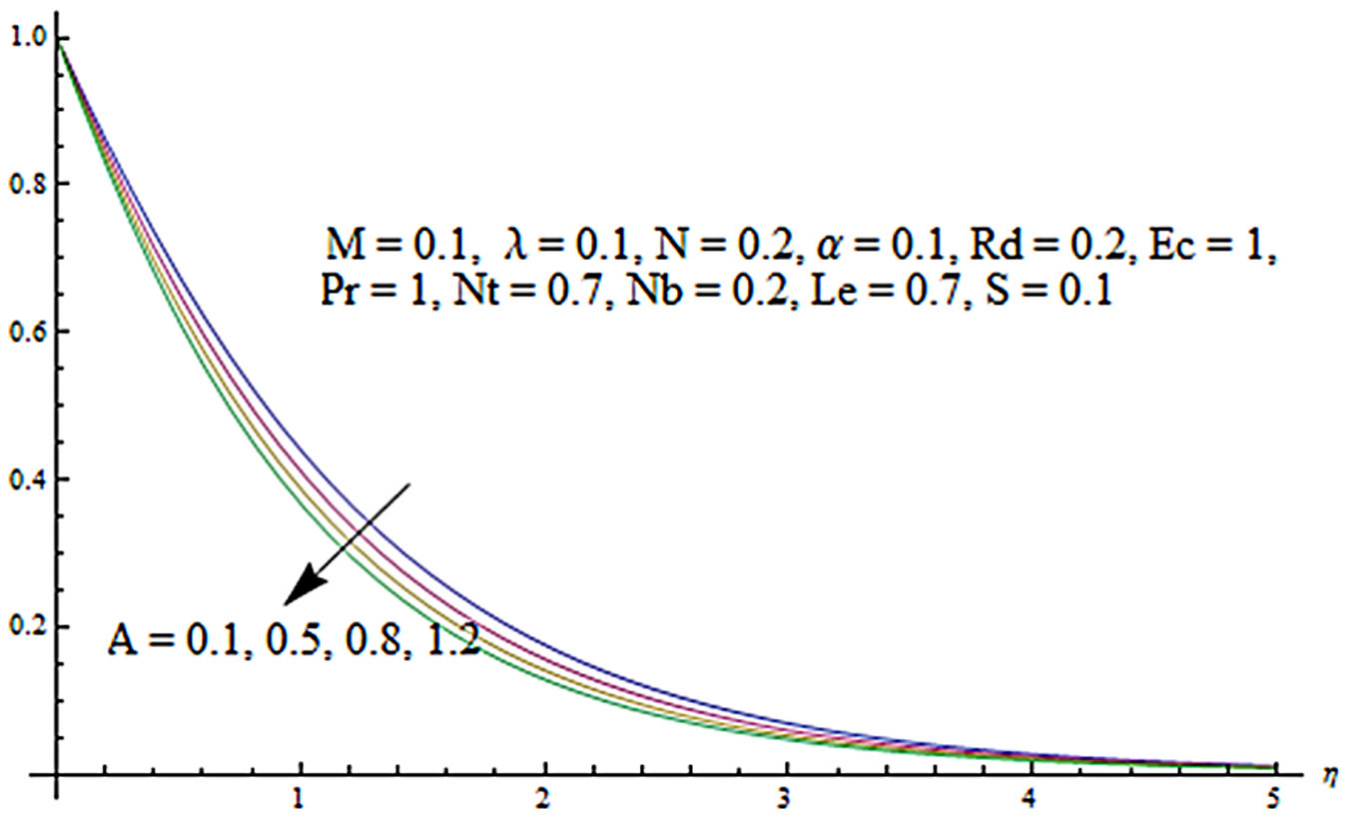
Influence of *A* on *θ*.

**Fig 4:**
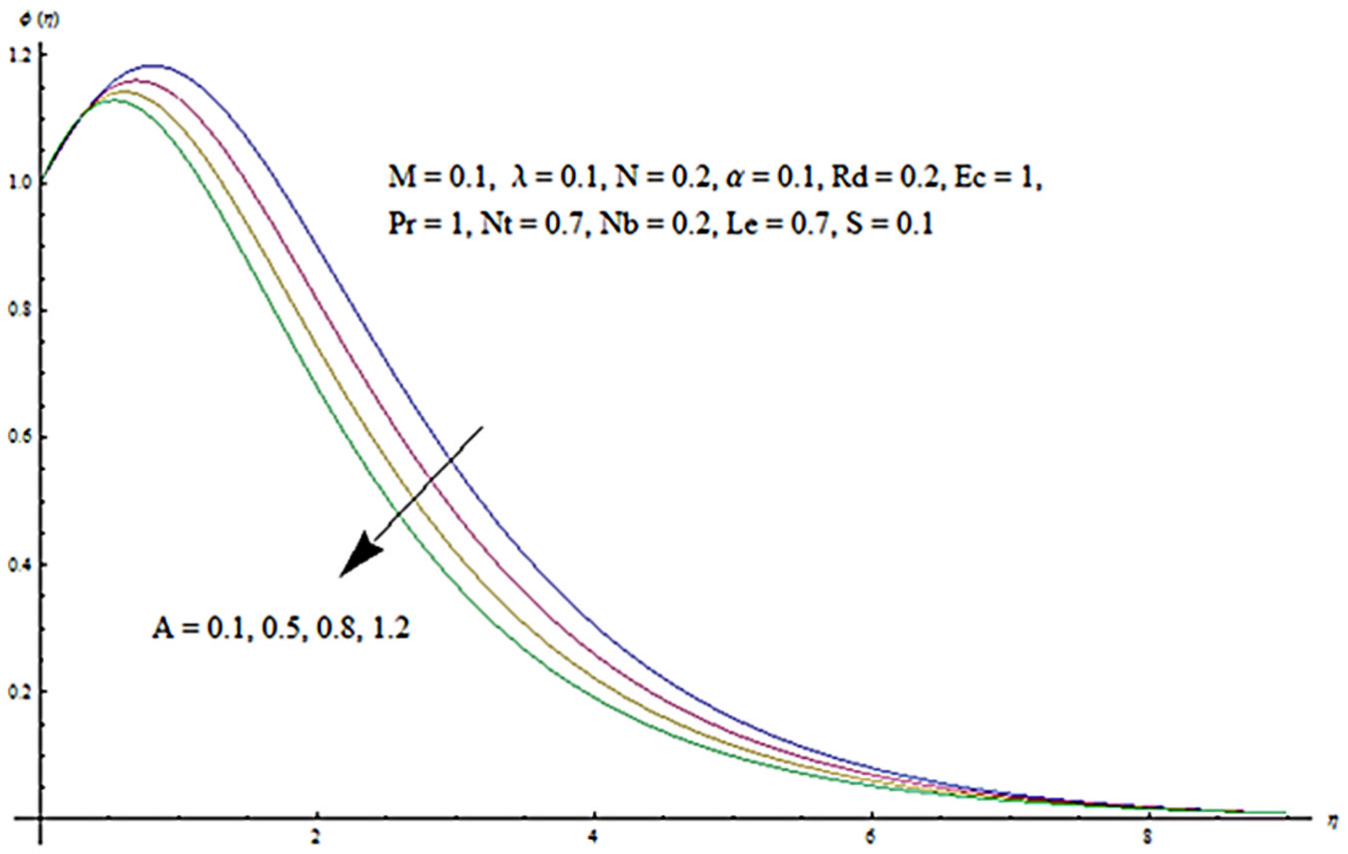
Influence of *A* on *ϕ*.

**Fig 5:**
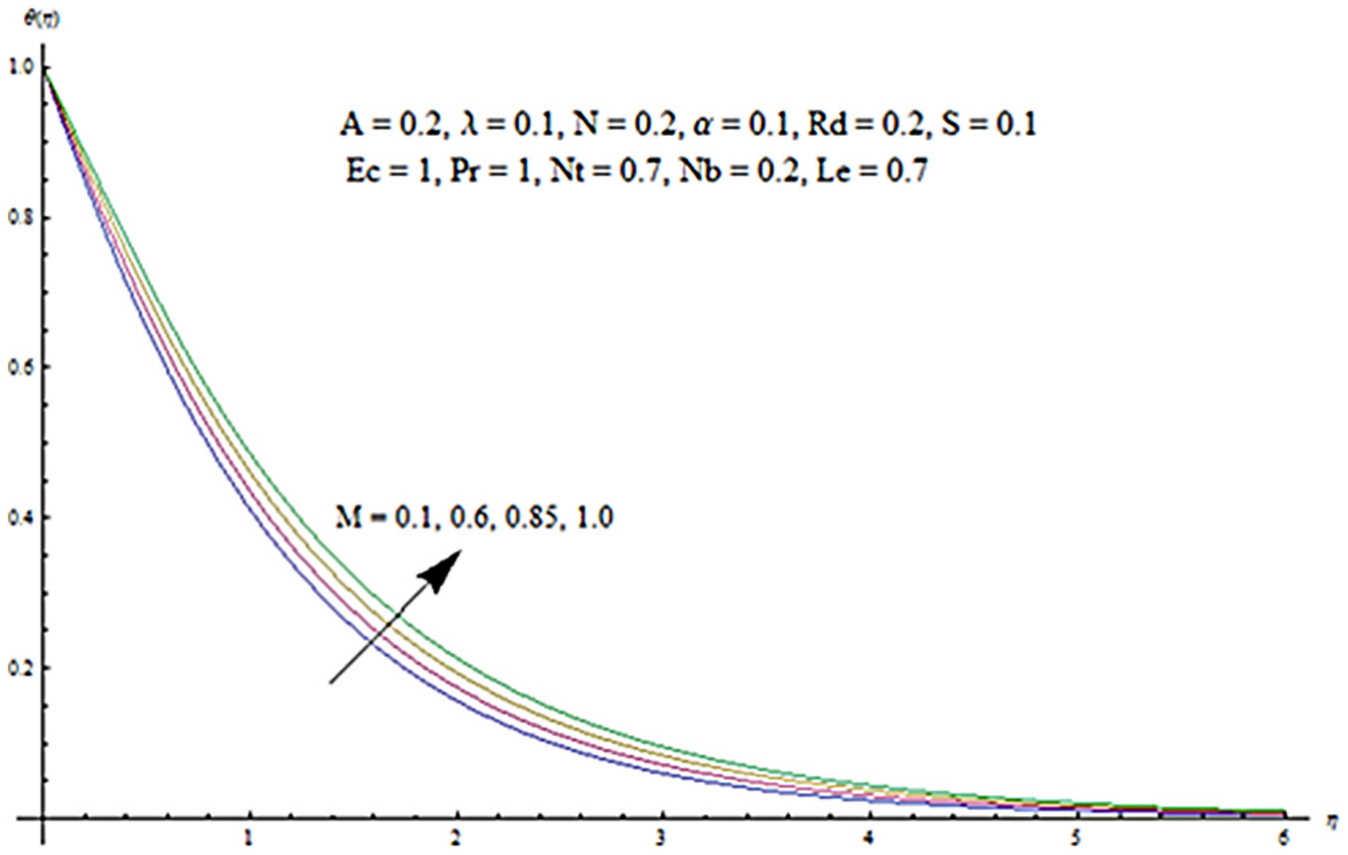
Influence of *M* on *θ*.

**Fig 6:**
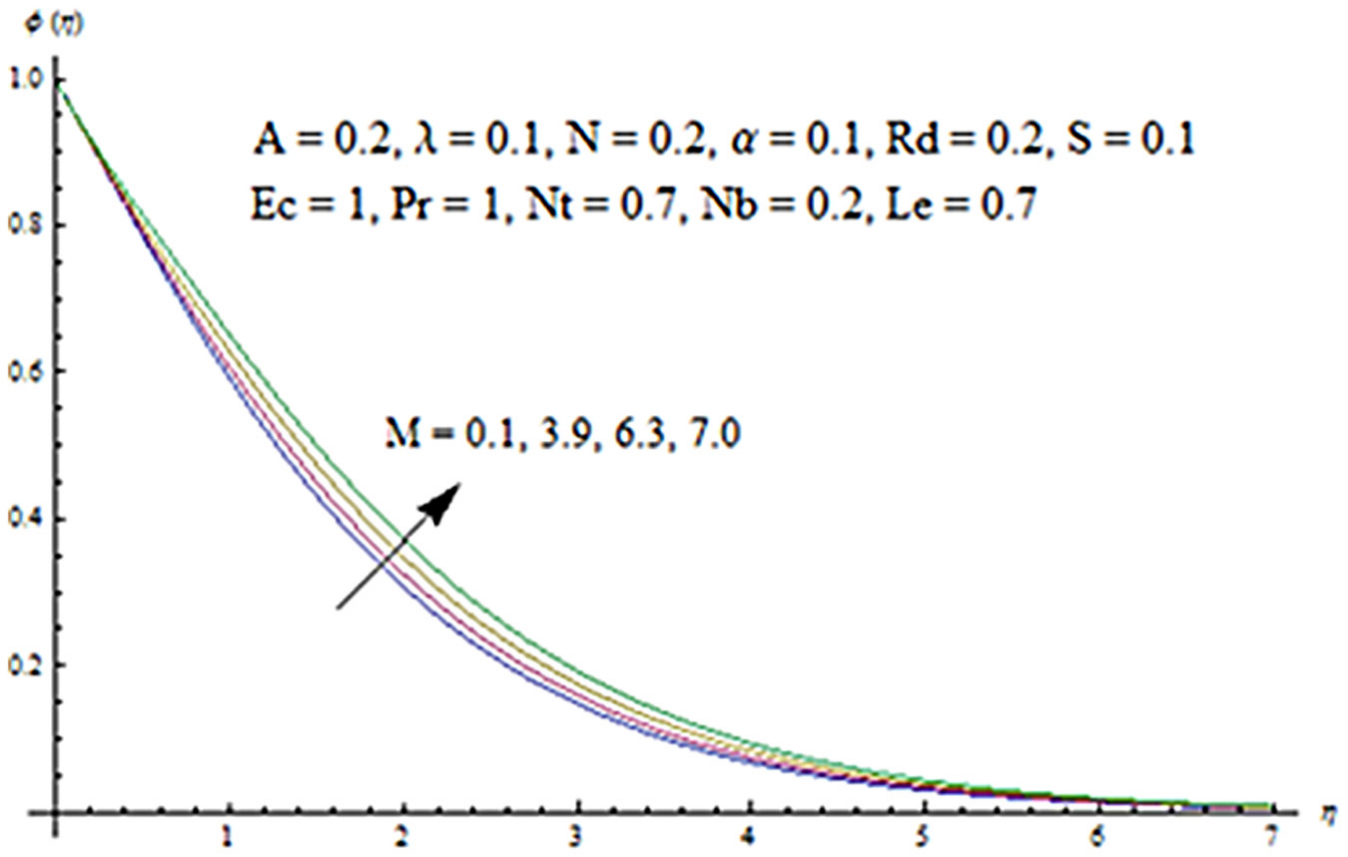
Influence of *M* on *ϕ*.

**Fig 7:**
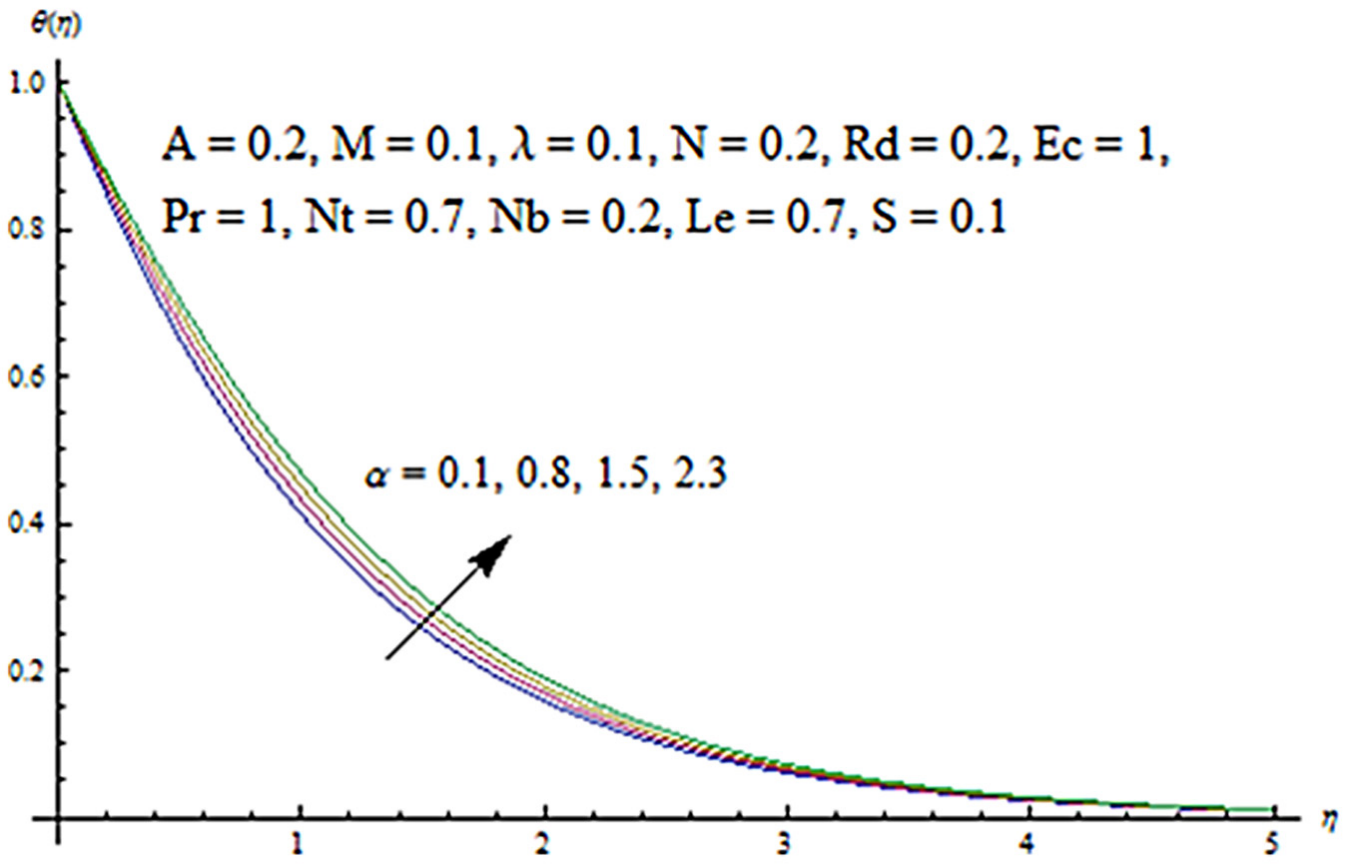
Influence of *α* on *θ*.

**Fig 8:**
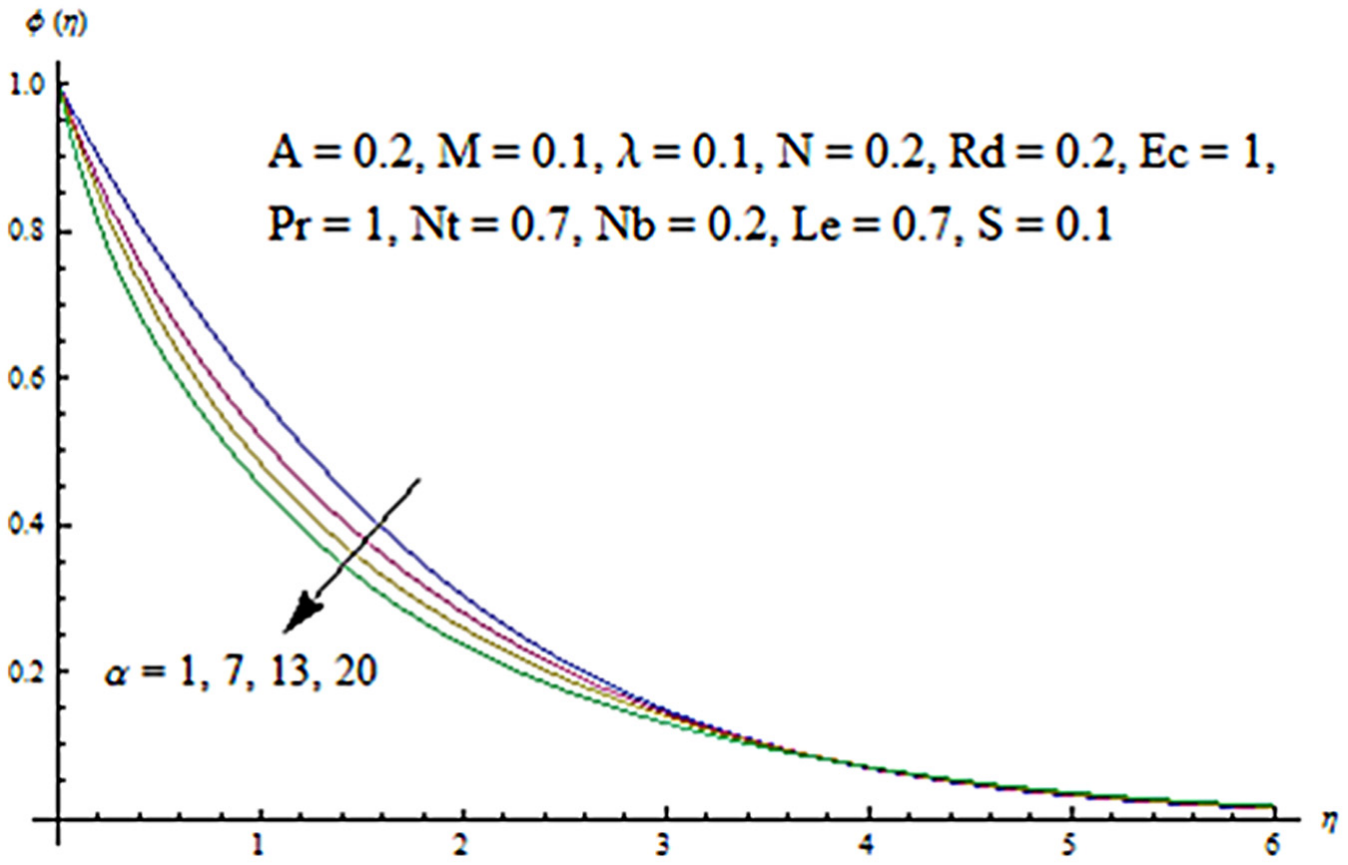
Influence of *α* on *ϕ*.

**Fig 9:**
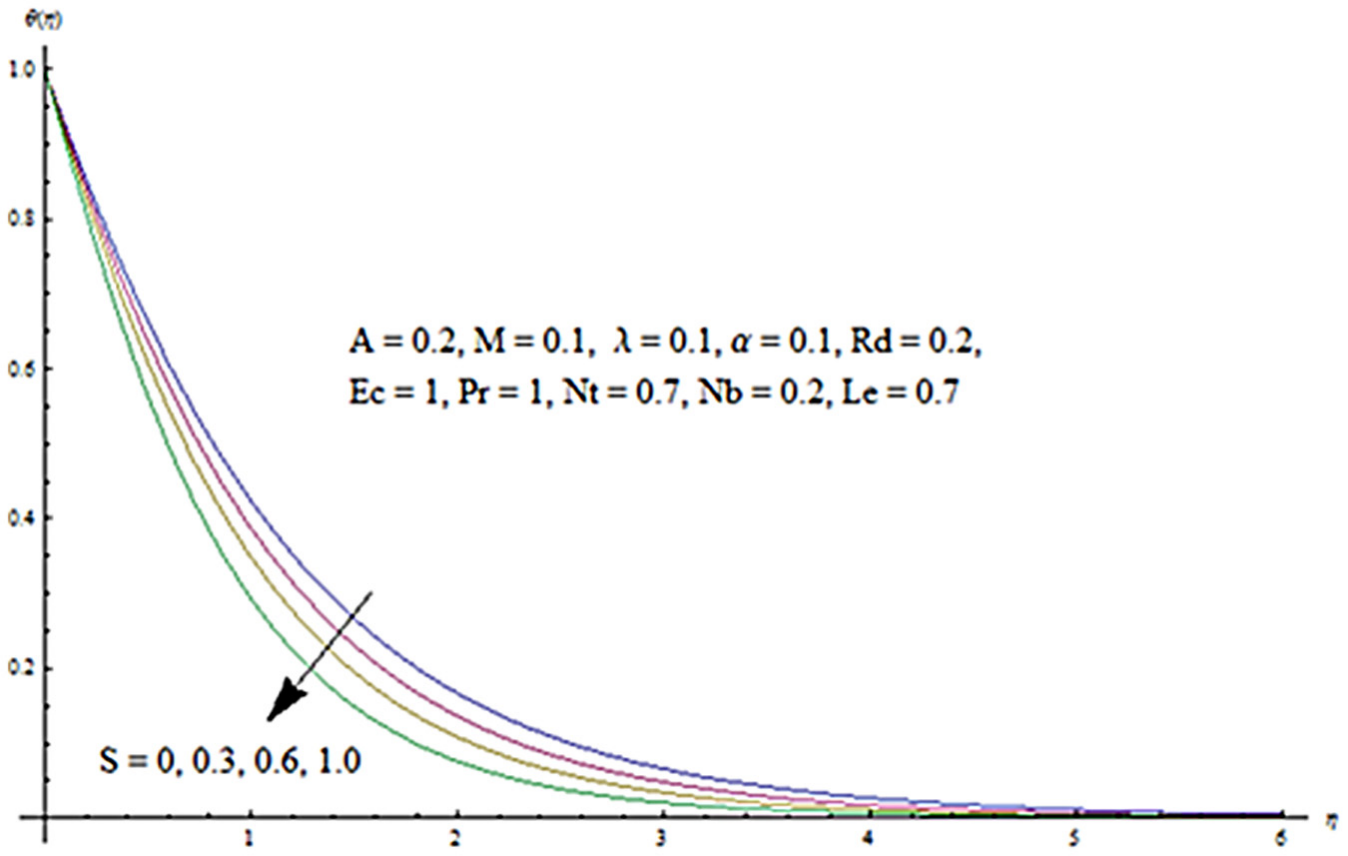
Influence of *S* on *θ*.

**Fig 10:**
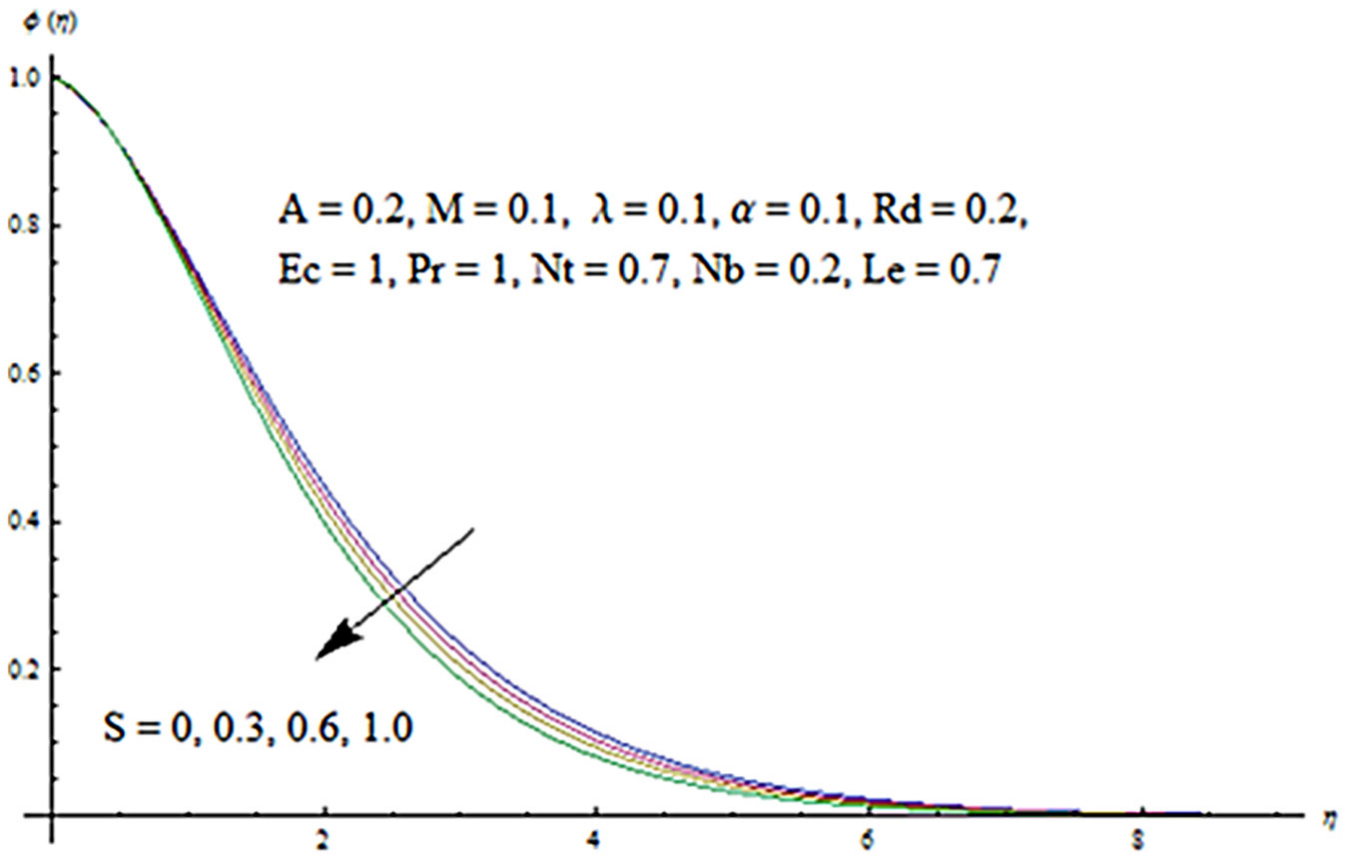
Influence of *S* on *ϕ*.

**Fig 11:**
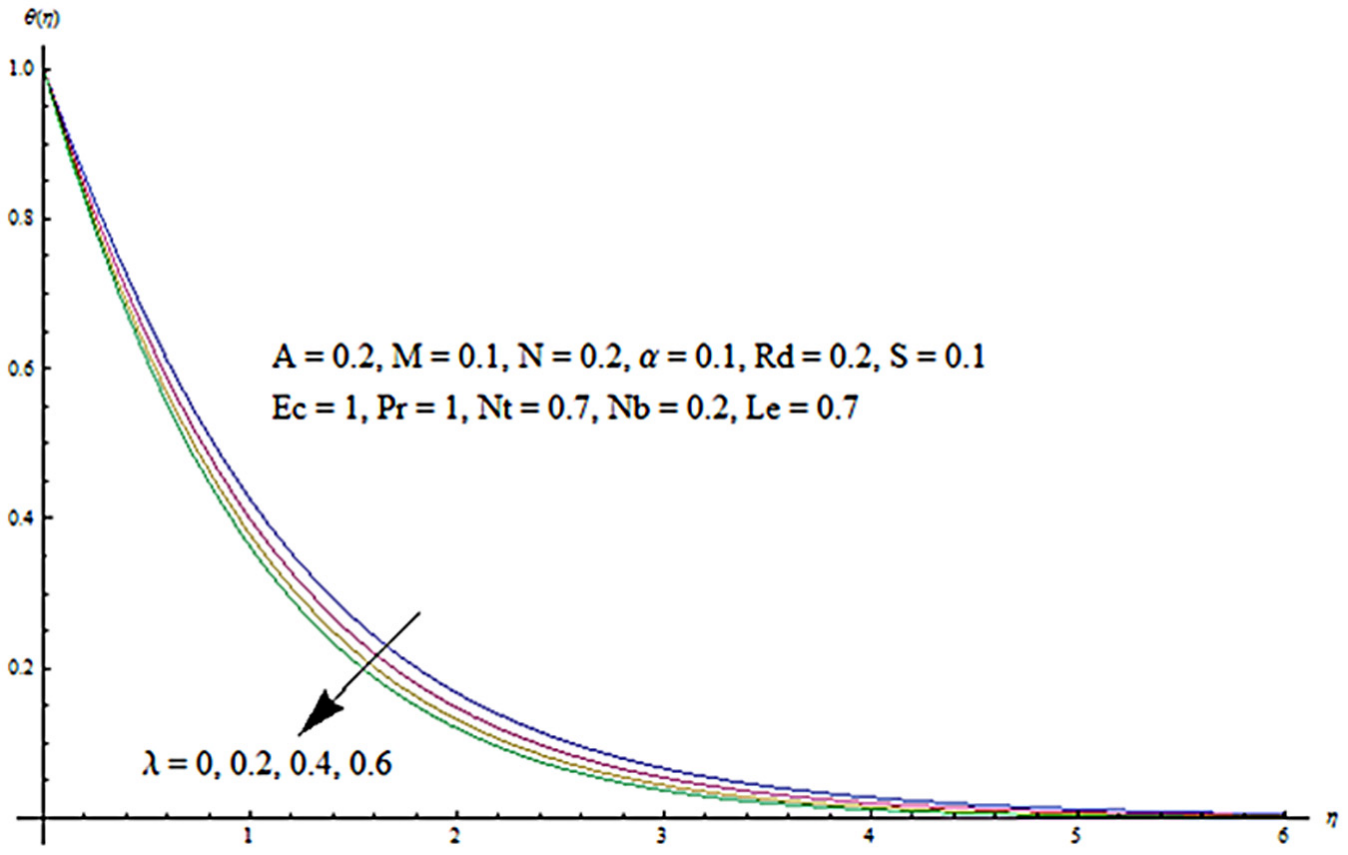
Influence of *λ* on *θ*.

**Fig 12:**
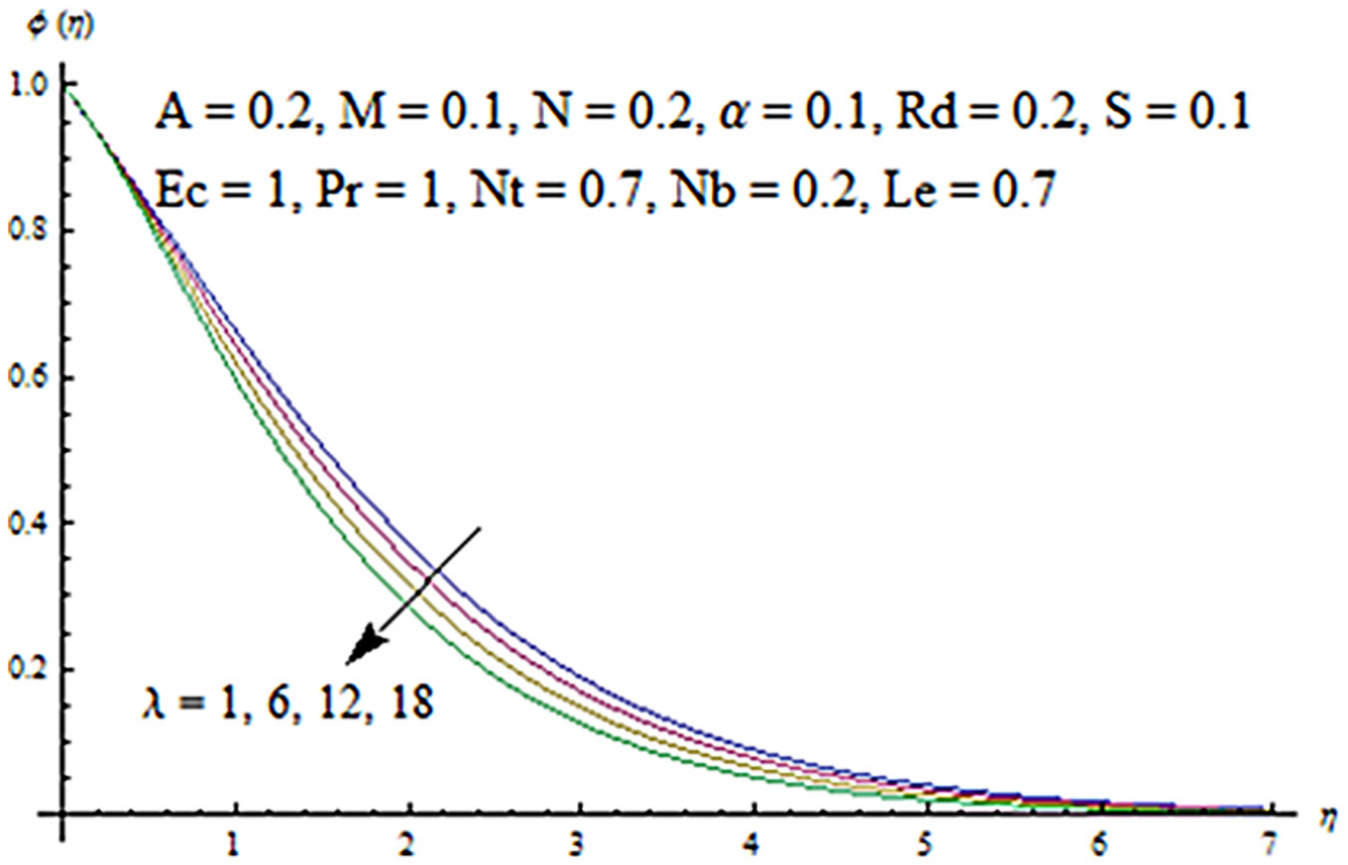
Influence of *λ* on *ϕ*.

Figs 3 and 4 presents the influence of unsteady parameter *A* on the temperature profile *θ* (*η*) and nanoparticle concentration *ϕ* (*η*). It is observed that an increase in unsteady parameter creates a reduction in the temperature and nanoparticle concentration profiles. Effects of Hartman number *M* on temperature and nanoparticle concentration fields are examined in Figs 5 and 6. Here we have observed that both temperature and nanoparticle concentration fields are enhanced with an increase in Hartman number. Physically, Hartman number involves the Lorentz force. This Lorentz force is stronger for the larger Hartman number due to which the temperature and nanoparticle concentration are increased. To examine the effects of second grade parameter *α* on the temperature and nanoparticle concentration profiles, we plotted the Figs 7 and 8. These figs. clearly show that an increase in second grade parameter gives rise to the temperature and thermal boundary layer thickness but a decrease is seen for the nanoparticle concentration profiles. Figs 9 and 10 depicts the variation in temperature and nanoparticle concentration profiles for different values of suction parameter *S*. An increase in suction parameter corresponds to a lower temperature and nanoparticle concentration profiles. Here suction parameter works as an agent which leads to a reduction in both temperature and nanoparticle concentration profiles. From Figs 11 and 12, we observe that both temperature and nanoparticle concentration fields are decreased when the values of buoyancy parameter *λ* are increased. It is due to the reason that buoyancy parameter has buoyancy force. This buoyancy force is stronger for larger buoyancy parameter. Such stronger buoyancy force leads a reduction in the temperature and nanoparticle concentration.

**Fig 13:**
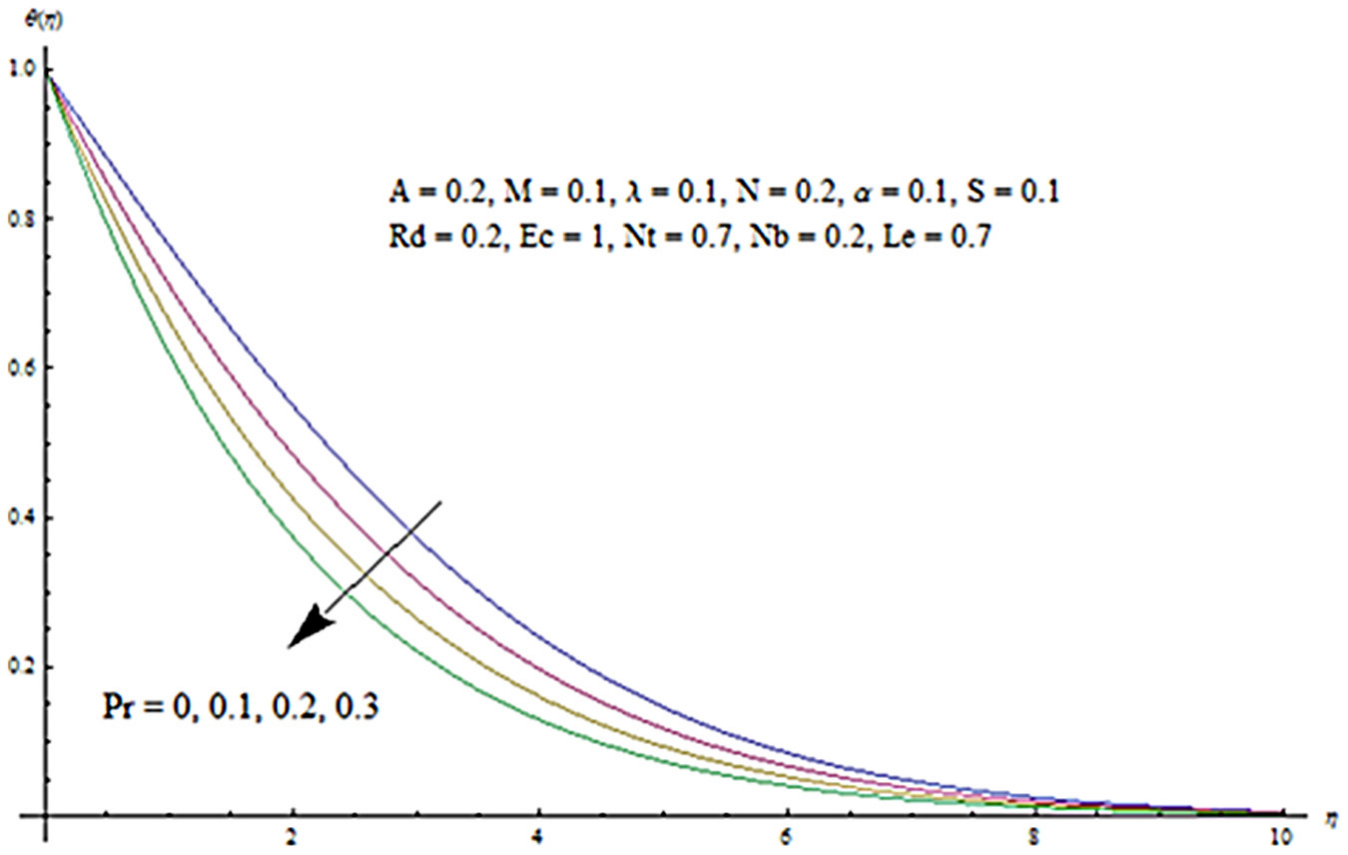
Influence of Pr on *θ*.

**Fig 14:**
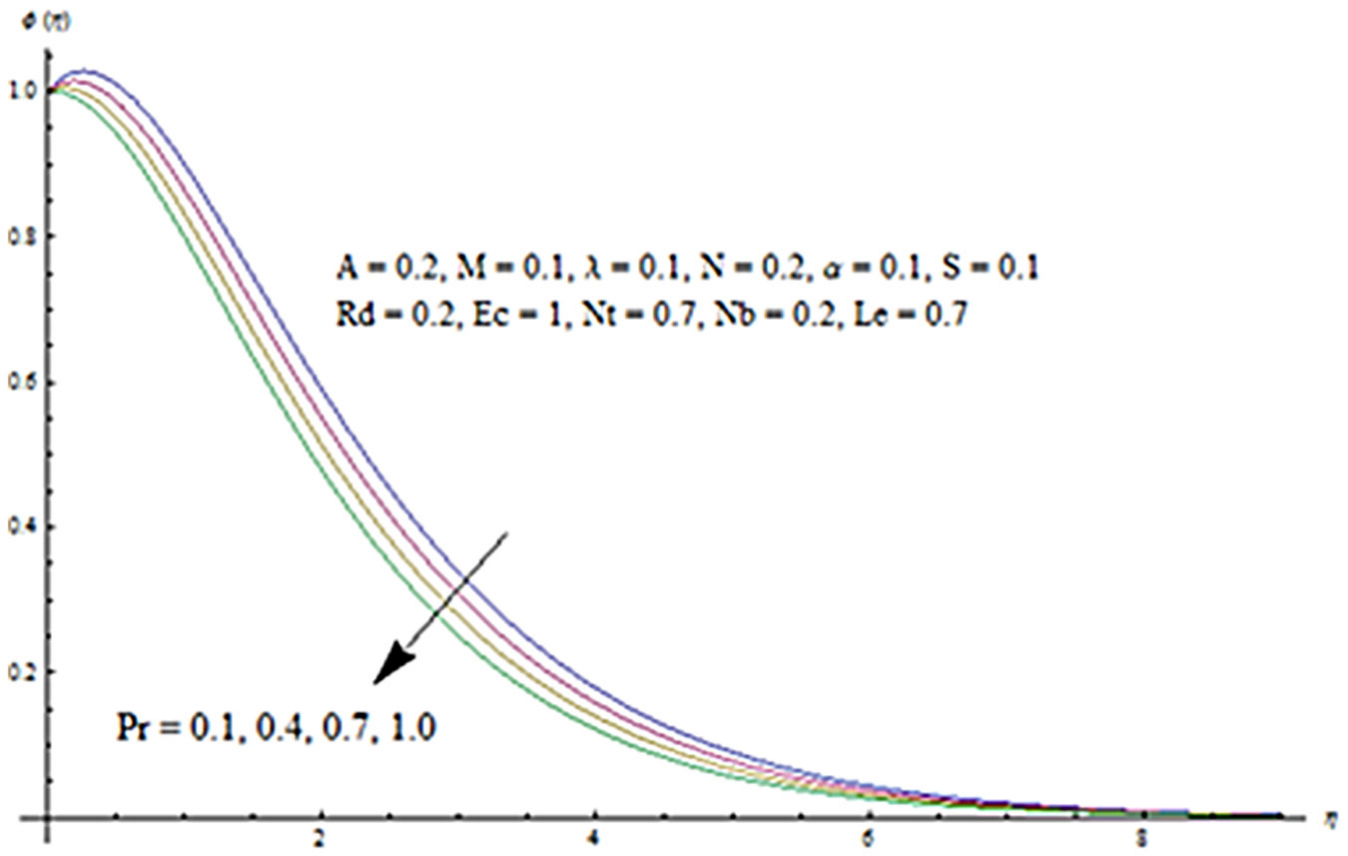
Influence of Pr on *ϕ*.

**Fig 15:**
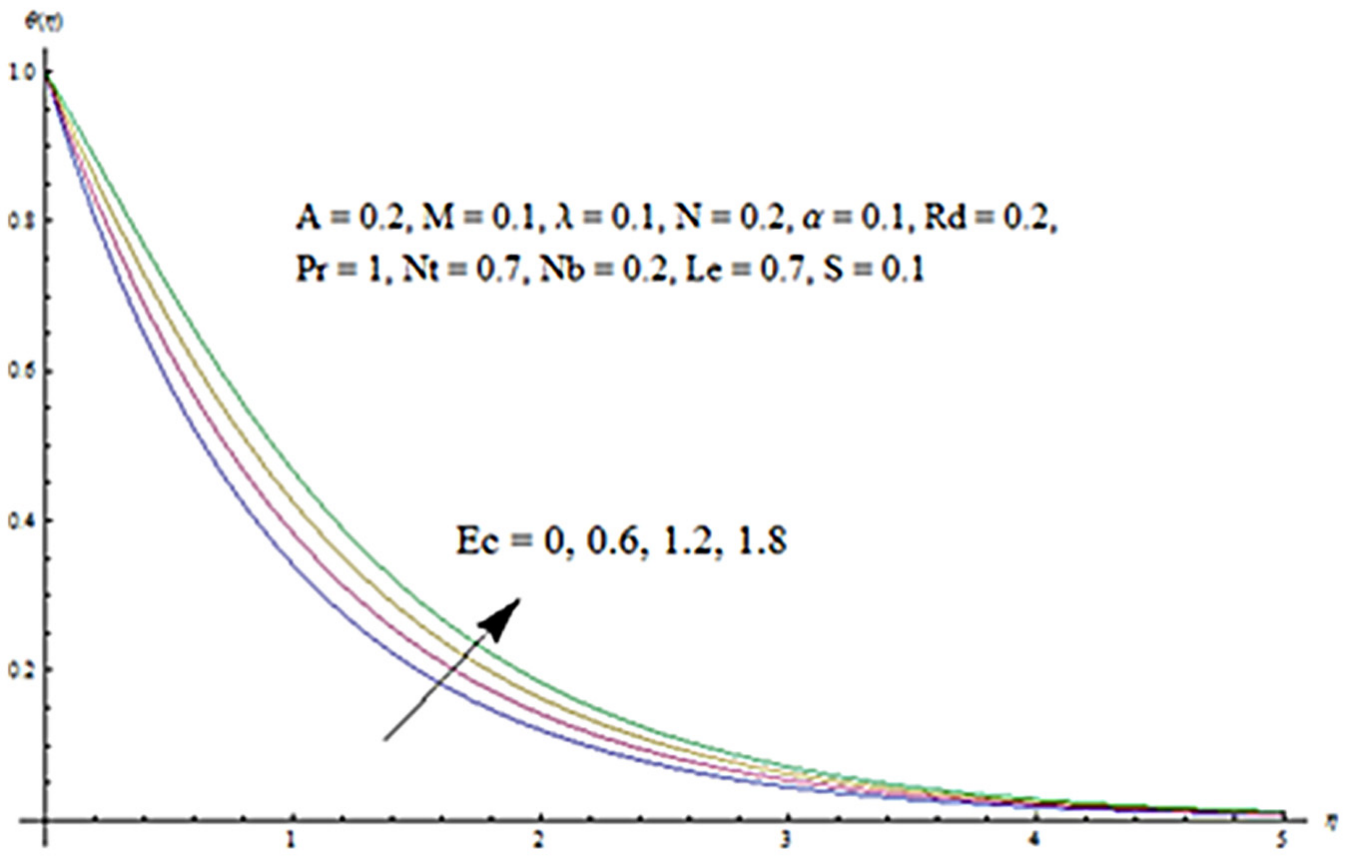
Influence of *Ec* on *θ*.

**Fig 16:**
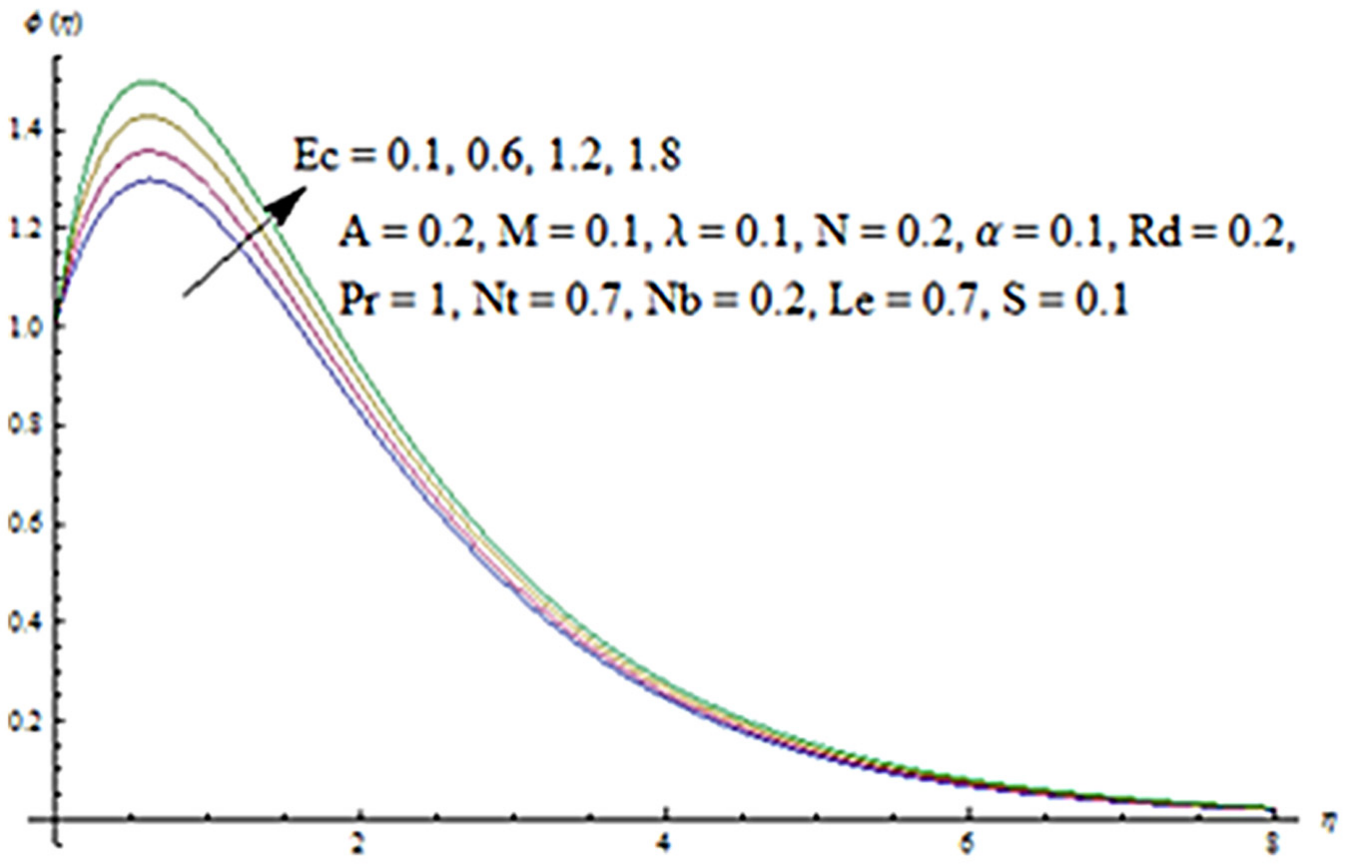
Influence of *Ec* on *ϕ*.

**Fig 17:**
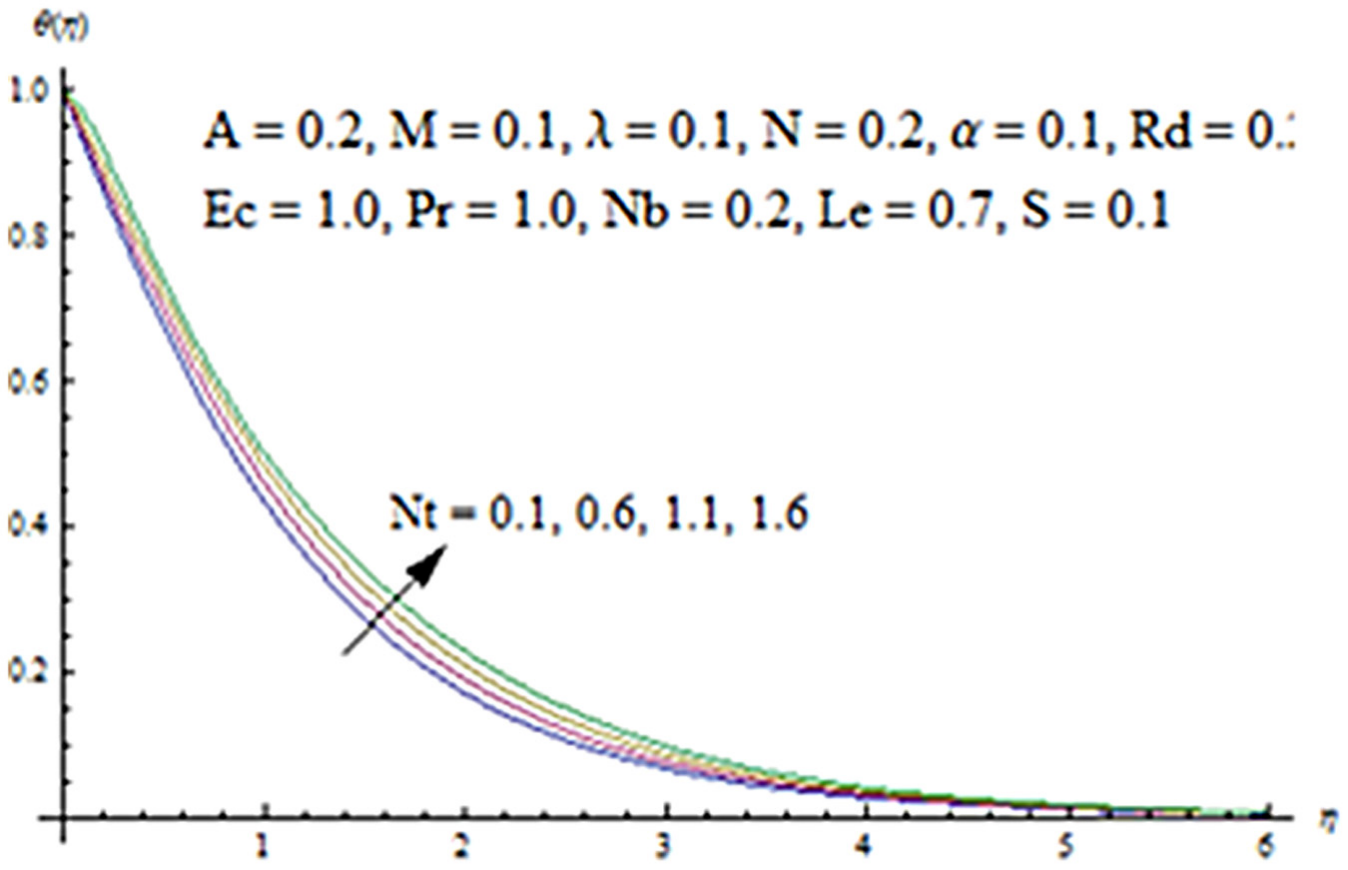
Influence of *Nt* on *θ*.

**Fig 18:**
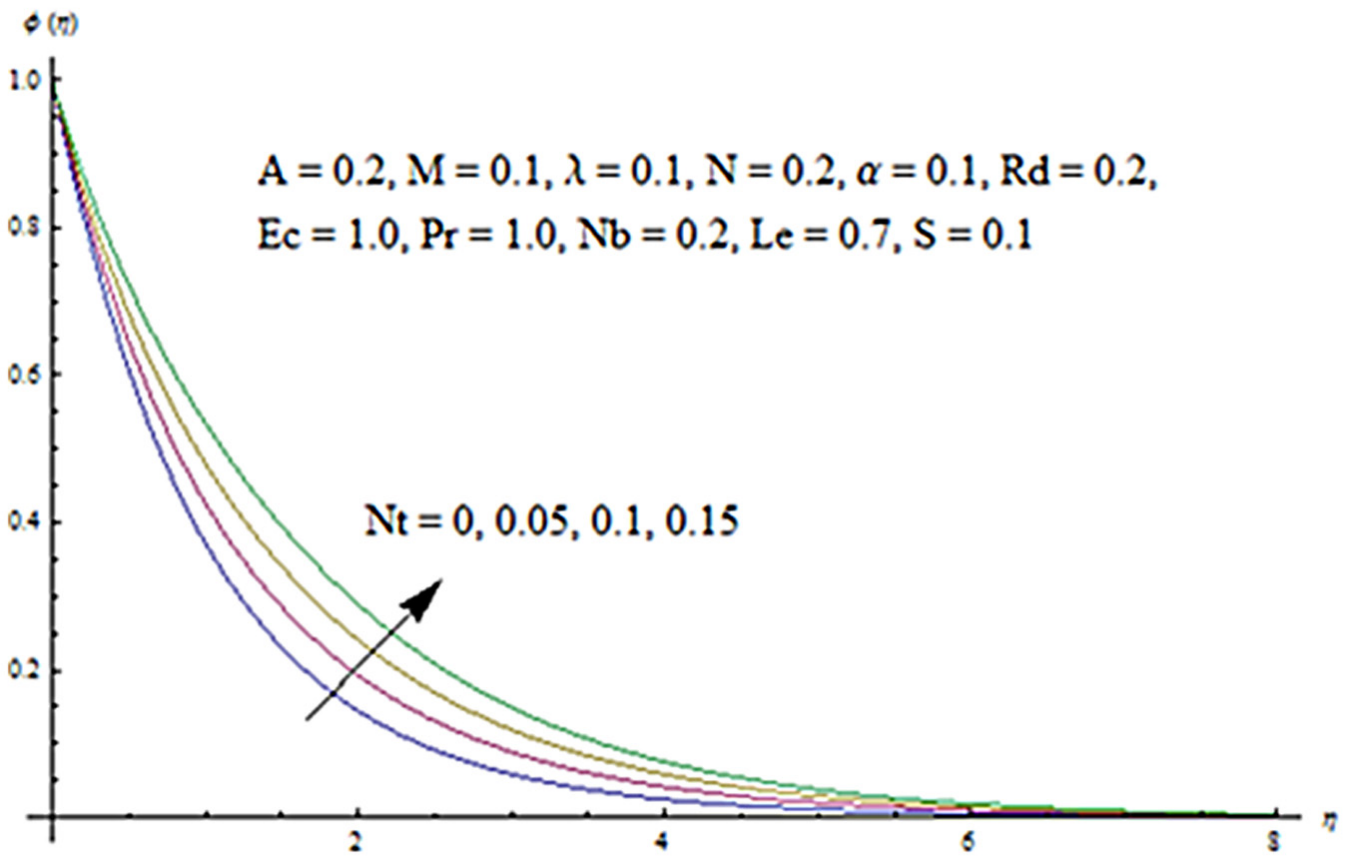
Influence of *Nt* on *ϕ*.

**Fig 19:**
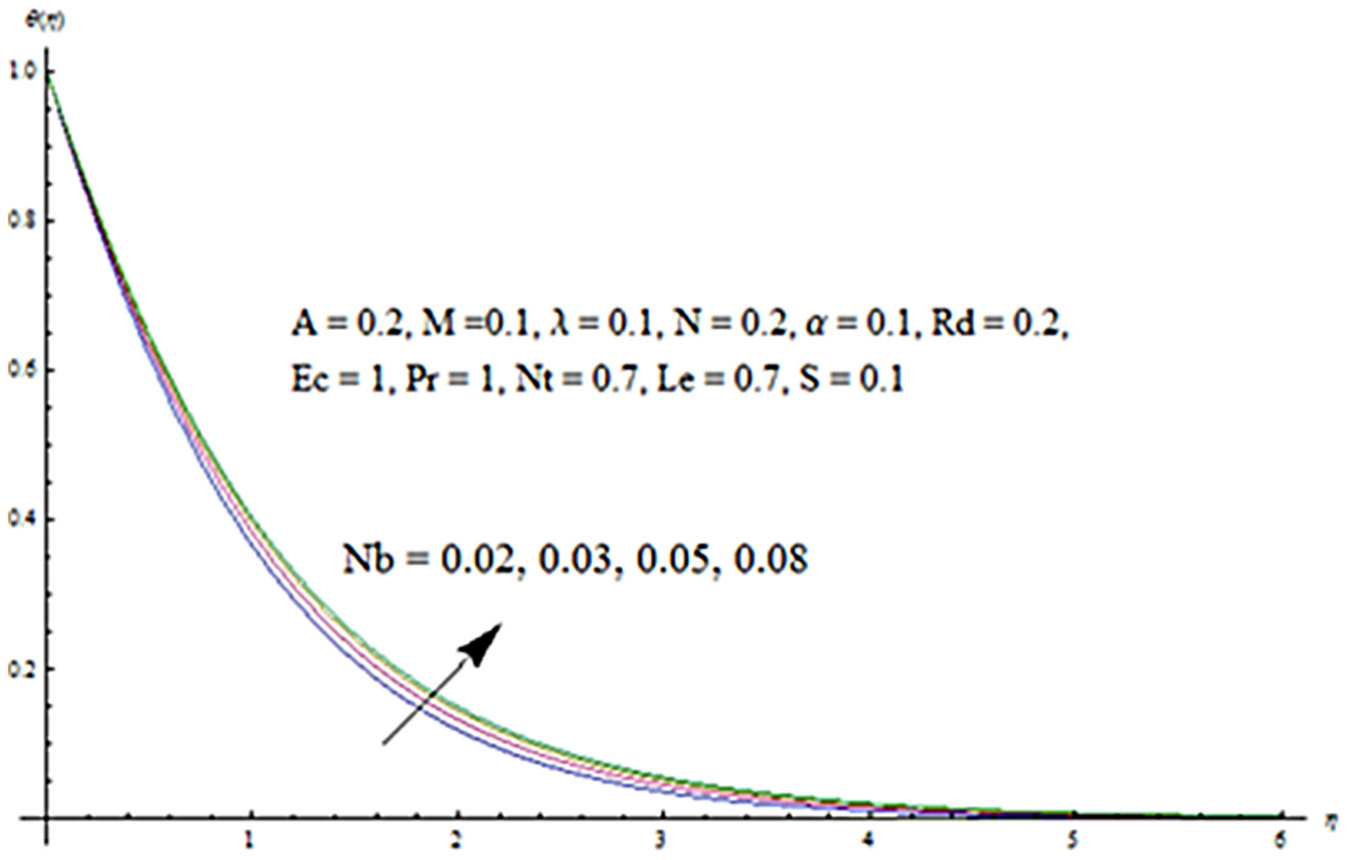
Influence of *Nb* on *θ*.

**Fig 20:**
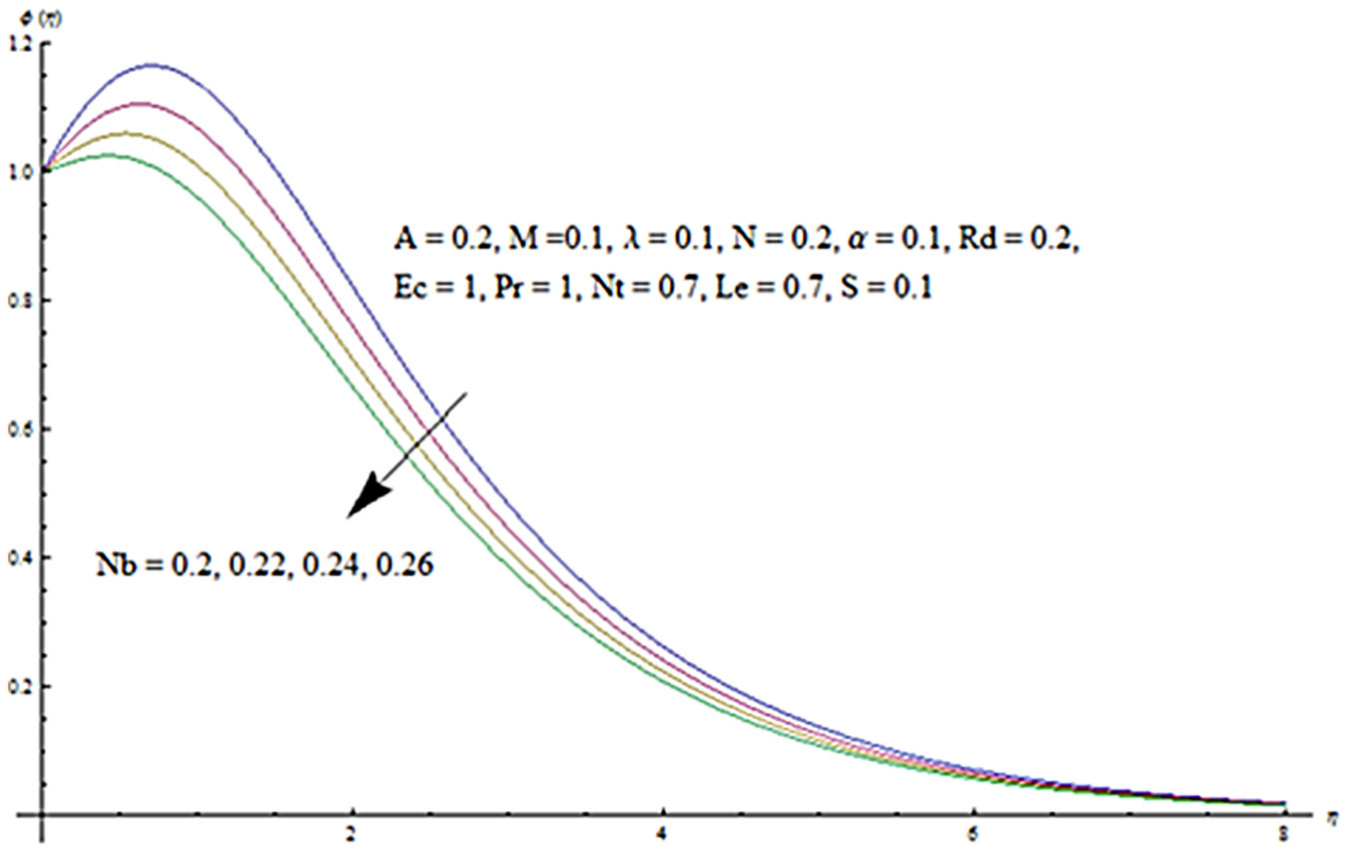
Influence of *Nb* on *ϕ*.

**Fig 21:**
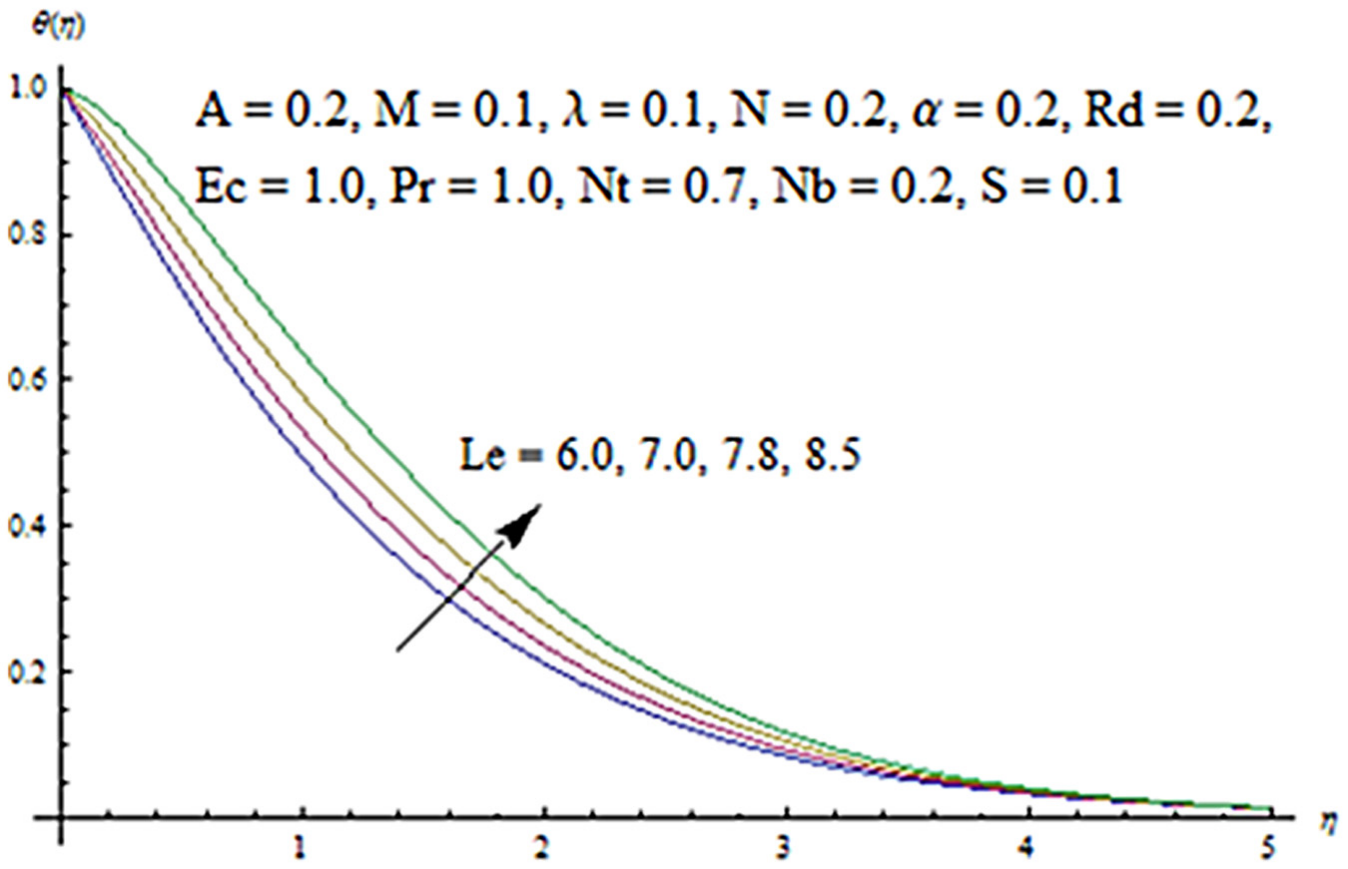
Influence of *Le* on *θ*.

**Fig 22:**
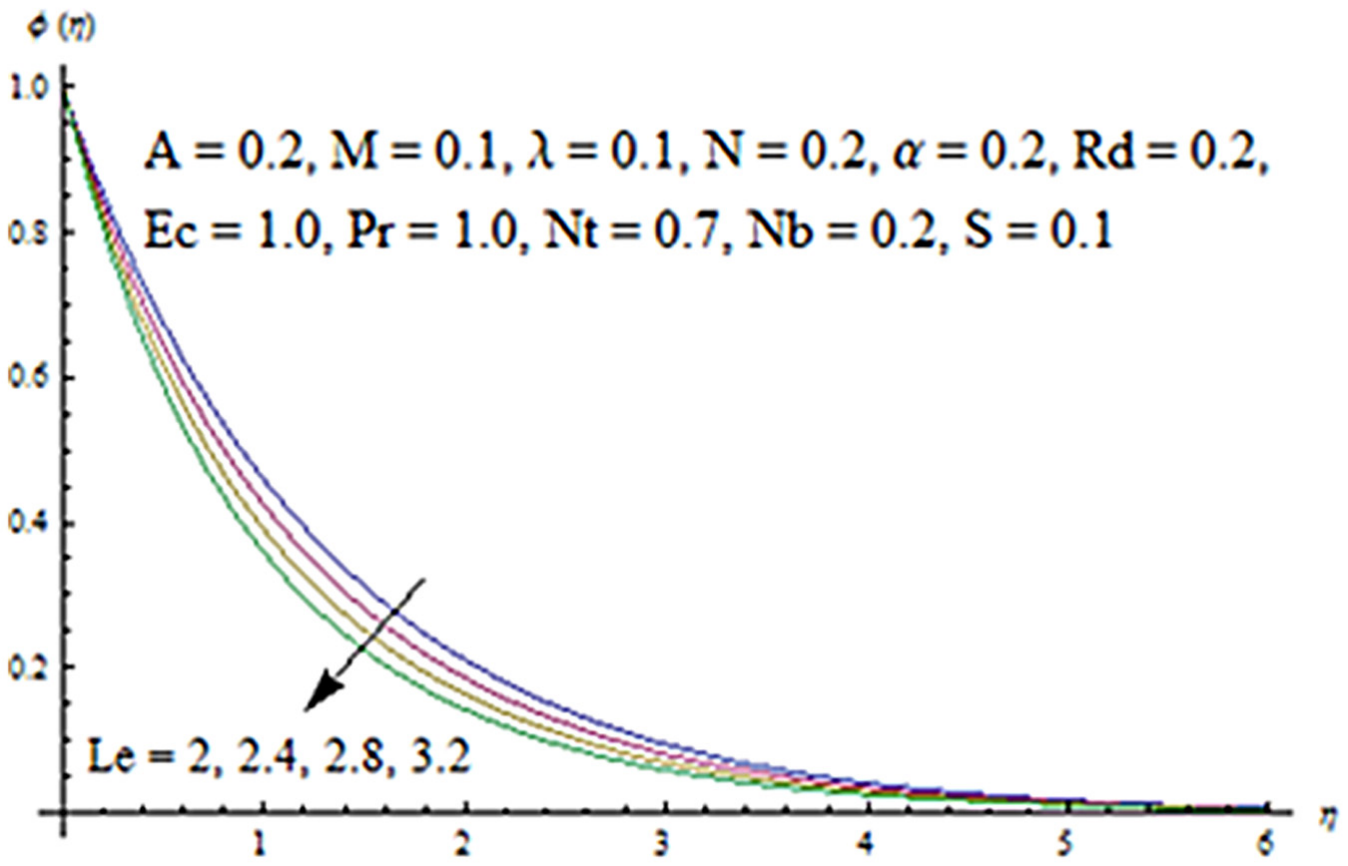
Influence of *Le* on *ϕ*.

**Fig 23:**
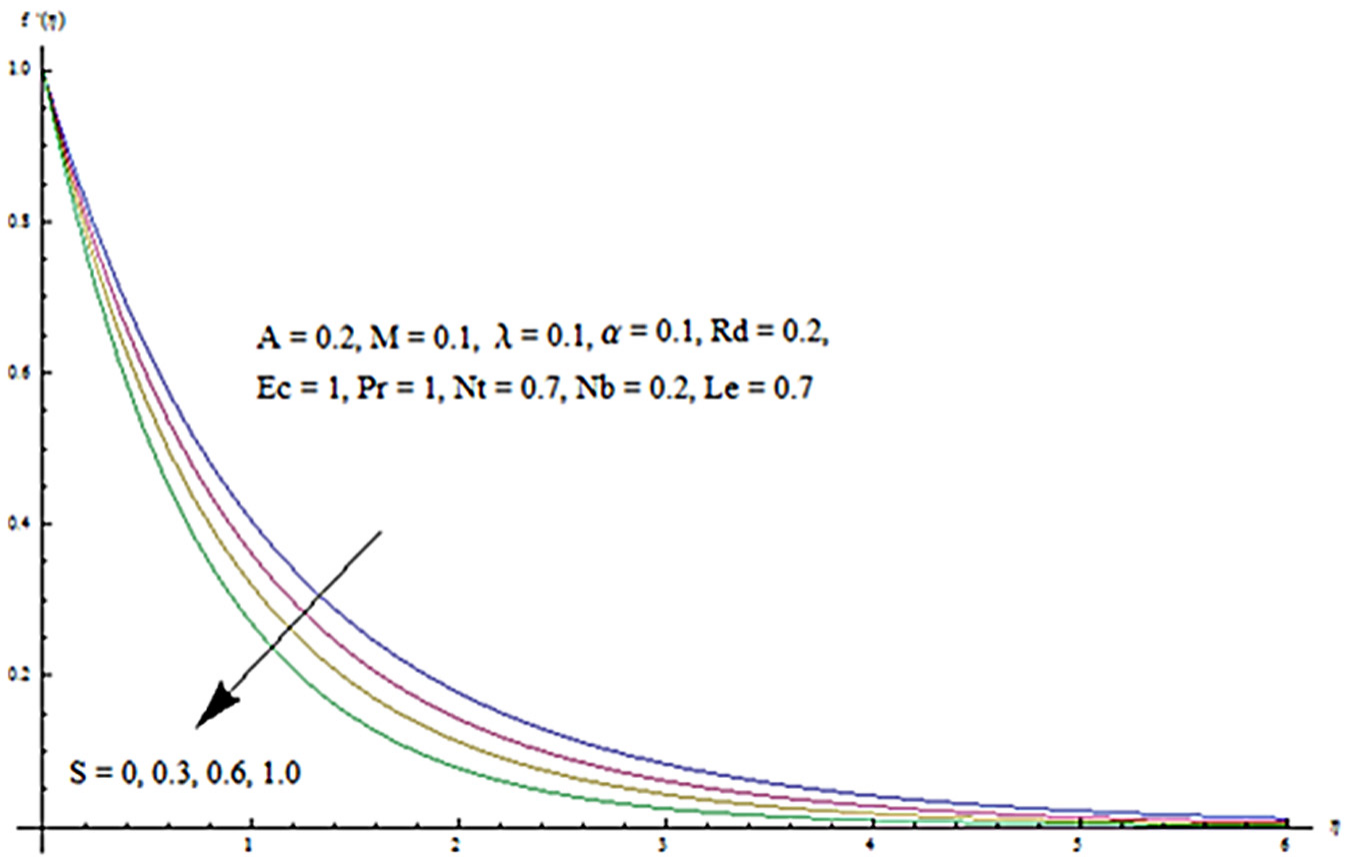
Influence of *S* on *f*′.

**Fig 24:**
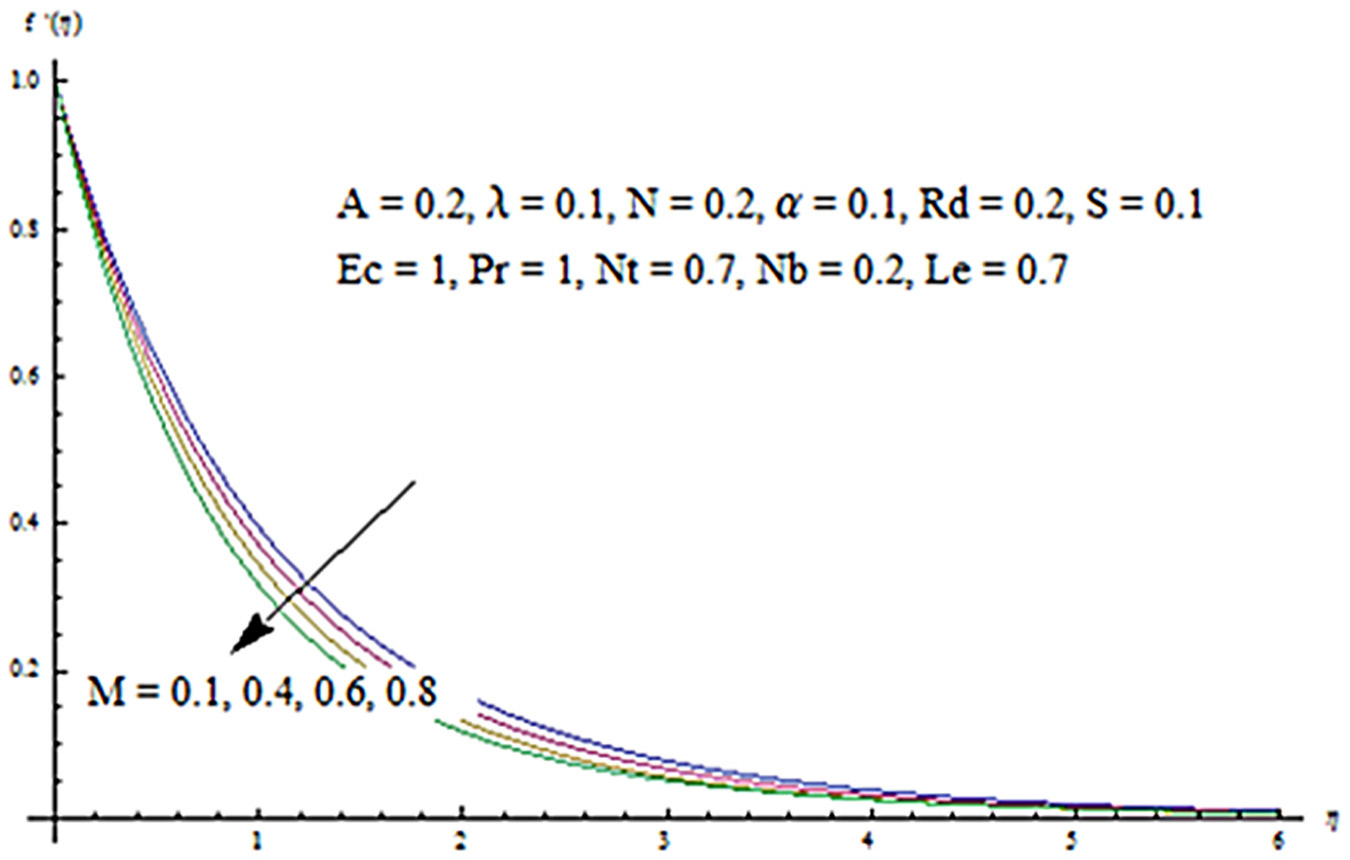
Influence of *M* on *f*′.

Figs 13 and 14 are presented to see the change in temperature and nanoparticle concentration corresponding to different values of *Pr*. From these figs. we analyzed that an increase in Prandtl number shows thinner thermal and nanoparticle concentration boundary layer thickness. Larger Prandtl number fluids have lower thermal diffusivity. Due to the lower thermal diffusivity, thinner thermal and nanoparticle concentration boundary layer thicknesses are observed. From Figs 15 and 16, it is seen that the larger values of Eckert number *Ec* corresponds to higher temperature and nanoparticle concentration. With an enhancement in value of Eckert number, we see an increase in kinetic energy due to which the temperature and nanoparticle concentration are enhanced. Influence of thermophoresis parameter *Nt* on temperature and nanoparticle concentration profiles is studied in the Figs 17 and 18. From these figs. we noted that an enhancement in thermophoresis parameter give rise to the temperature and nanoparticle concentration profiles. The variations in nanoparticle concentration profile are more pronounced in comparison to the temperature field due to an increase in thermophoresis parameter. Figs 19 and 20 illustrate that both temperature and nanoparticle concentration profiles are quit opposite due to an enhancement in Brownian motion parameter. An increase in Lewis number *Le* shows an increase in temperature and decrease in nanoparticle concentration profile. (see Figs 21 and 22). The variation of suction parameter *S* on velocity *f*′ is shown in Fig 23. It is observed that velocity and boundary layer thickness decrease with an increase in values of *S*. In practical, the porosity of wall controls the boundary layer flow. Fig 24 depicts that *f*′ is a decreasing function of *M* because increasing the value of *M* results in an increase in Lorentz force thus decrease the magnitude of velocity.

Table 1 gives the convergent values of *f*″(0), *θ*′(0) and *ϕ*′(0) at different order of HAM deformations. Here it is seen that the values of *f*″(0) converges from 10-th order of deformations while the values of *θ*′(0) and *ϕ*′(0) repeats from 13-th and 16-th order of computations. Hence the 16-th order of HAM computations is essentials for the convergent homotopic solutions. Table 2 presents the numerical values of skin-friction coefficient, local Nusselt and Sherwood numbers for different values of *A*, *M*, *λ*, *N*, *α* and *Rd* when *Pr* = 1 = *Ec*, *Nt* = 0.7, *Nb* = 0.2, *Le* = 0.7 and *S* = 0.1. From this table, it is examined that the values of skin-friction coefficient are increased with an increase in *N* but the values of Nusselt and Sherwood number are reduced. The values of Skin-friction coefficient, local Nusselt and Sherwood numbers for different values of *Pr*, *Nb*, *Nt*, *Le* and *S* when *A* = 0.2, *M* = 0.1, *λ* = 0.1, *α* = 0.1, *N* = 0.2 and *Rd* = 0.2 are studied in Table 3. The numerical values of skin-friction coefficient, local Nusselt and Sherwood numbers are enhanced with an increase in the value of *Pr*. The Nomenclature of all parameters used is depicted in Table 4.

**Table 1:** Convergence of series solutions for different order of approximations when *A* = 0.2, *M* = 0.1, *λ* = 0.1, *N* = 0.2, *α* = 0.1, *Rd* = 0.2, *Ec* = 1.0, *Pr* = 1.0, *Nt* = 0.7, *Nb* = 0.2, *Le* = 0.7, *S* = 0.1 and for ℏ = −0.7.

Order of approximations	−*f*″(0)	−*θ*′(0)	−*ϕ*′(0)
1	0.99800	0.77022	0.39400
4	0.97740	0.78347	0.35521
6	0.97560	0.78815	0.40701
10	0.97500	0.78986	0.42402
13	0.97498	0.78993	0.42438
16	0.97498	0.78993	0.42413
25	0.97498	0.78993	0.42412

**Table 2:** Numerical values of skin friction coefficient $C_{f}{\text{Re}}_{x}^{1/2}$, local Nusselt number $Nu{\text{Re}}_{x}^{-1/2}$ and sherwood number $Sh{\text{Re}}_{x}^{-1/2}$ for different parameters when *Pr* = 1 = *Ec*, *Nt* = 0.7, *Nb* = 0.2, *Le* = 0.7 and *S* = 0.1.

*A*	*M*	*λ*	*N*	*α*	*Rd*	-$C_{f}{\text{Re}}_{z}^{1/2}$	-$Nu{\text{Re}}_{z}^{-1/2}$	-$Sh{\text{Re}}_{z}^{-1/2}$
0.2	0.1	0.1	0.2	0.1	0.2	1.30339	0.65362	0.04239
0.1						1.25073	0.59409	0.06537
0.2						1.30339	0.65362	0.04239
0.3						1.35471	0.70814	0.03112
	0.2					1.32151	0.64652	0.02728
	0.3					1.35117	0.63492	0.03191
	0.4					1.39169	0.61901	0.03272
		0.1				1.30339	0.65362	0.04239
		0.2				1.21428	0.69074	0.11761
		0.3				1.12988	0.72281	0.18316
			0.1			1.31660	0.64633	0.02989
			0.2			1.30339	0.65362	0.04239
			0.3			1.29044	0.66062	0.05457
				0.1		1.30339	0.65362	0.04239
				0.2		1.53602	0.65094	0.01518
				0.3		1.74899	0.64792	0.01032
					0.1	1.30496	0.67833	0.09838
					0.2	1.30339	0.65362	0.04239
					0.3	1.30192	0.63123	0.00784

**Table 3:** Numerical values of skin friction coefficient $C_{f}{\text{Re}}_{x}^{1/2}$, local Nusselt number $Nu{\text{Re}}_{x}^{-1/2}$ and sherwood number $Sh{\text{Re}}_{x}^{-1/2}$ for different parameters when *A* = 0.2, *M* = 0.1, *λ* = 0.1, *α* = 0.1, *N* = 0.2 and *Rd* = 0.2.

*Pr*	*Nt*	*Nb*	*Le*	*S*	*Ec*	-$C_{f}{\text{Re}}_{z}^{1/2}$	-$Nu{\text{Re}}_{z}^{-1/2}$	-$Sh{\text{Re}}_{z}^{-1/2}$
0.5						1.28232	0.51721	0.27114
0.8						1.28679	0.61215	0.15009
1.2						1.30856	0.68367	0.07743
	0.5					1.30846	0.67376	0.21774
	0.7					1.30339	0.65362	0.04239
	0.9					1.29856	0.63442	0.02745
	1.0					1.29621	0.62517	0.02612
		0.1				1.28926	0.68457	1.25400
		0.2				1.30339	0.65362	0.042416
		0.3				1.30783	0.62998	0.035137
			0.5			1.29825	0.66993	0.38878
			0.7			1.30339	0.65362	0.04239
			0.9			1.30687	0.64212	0.02374
				0.2		1.36652	0.66523	0.06457
				0.3		1.43250	0.67692	0.08701
				0.4		1.50128	0.68871	0.10960
					0.5	1.30572	0.78782	0.44115
					1.0	1.30339	0.65362	0.04239
					1.5	1.30107	0.51990	0.035497

**Table 4:** Nomenclature.

Nomenclature			
B(t)	Magnetic field	T	Fluid temperature
(u, v)	Velocity components	T_∞_	Ambient temperature
*μ*	Dynamic viscosity	C_∞_	Ambient concentration
*υ*	Kinematic viscosity	*βc*	Concentration coefficient
*ρ*	Fluid Density	Pr	Prandtl number
*α*_1_, *α*_2_	Second grade parameters	Ec	Eckert number
σ*	Stefan-Boltzmann constant	*λ*	mixed convection parameter
Cp	Specific heat	q_*r*_	radiative heat flux
*κ*	Thermal conductivity	Le	Lewis number
*β*_*T*_	Thermal expansion coefficient	C_*f*_	Skin friction
*σ*	Electrical conductivity	*τ*_*w*_	Wall shear stress
q_*w*_	Surface heat flux	j_*w*_	Mass flux
A	Unsteady parameter	Nu_*x*_	Nusselt number
g	Gravitational acceleration	q_*w*_	Surface heat flux
G_*r*_*x*__	Grashof number	R_*d*_	radiation parameter
N_*B*_	Brownian motion parameter	N_*T*_	Thermophoresis parameter
D_*B*_	Brownian diffusion coefficient	Re_*x*_	Local Reynolds number
D_*T*_	Thermophoretic diffusion coefficient	*ψ*	Stream function
κ*	Mean absorption coefficient	*η*	Dimensionless variable
U_*W*_	Stretching surface velocity	f	Dimensionless stream function
*θ*	Dimensionless temperature	*ϕ*	Dimensionless concentration
*L*_*f*_	Linear operator for momentum	*L*_*θ*_	Linear operator for energy
*L*_*ϕ*_	Linear operator for concentration	ℏ_*θ*_	Auxiliary parameter for energy
ℏ_*f*_	Auxiliary parameter for momentum	ℏ_*ϕ*_	Auxiliary parameter for concentration
*S*	Suction/injection parameter	a, c	Dimensional constants
T_*w*_	Wall temperature	*M*	Hartman number
*Sh*	Sherwood number	V_*w*_	Suction /injection velocity
t	time	C_*w*_	Wall concentration

## Final remarks

Unsteady MHD flow of second grade nanofluid induced by vertical sheet with mixed convection and thermal radiation is examined by Homotopy analysis method. The behavior of arised parameters have been discussed. The salient features of this exploration are appended below.

- The influence of Hartman number *M*, second grade dimensionless parameter *α* and Eckert number *Ec* on *θ* are similar.
- An increase in second grade parameter results in an increase in temperature and thermal boundary layer thickness but a decrease is seen for the nanoparticle concentration profiles
- Thermal boundary layer thickness and temperature *θ* (*η*) decrease by increasing buoyancy parameter *λ*.
- *Nt* and *Nb* are increasing functions of the temperature *θ* (*η*) whereas they depict an opposite behavior in case of Concentration *ϕ*.
- Pr decreases with an increase in values of temperature *θ* and concentration *ϕ*.
- For increasing values of *λ*, Skin friction coefficient and local Nusselt number increase whereas sherwood number decreases.
- *Le* show an opposite behavior for temperature *θ* and concentration *ϕ*.
